# The Phylogeny of Class B Flavoprotein Monooxygenases and the Origin of the YUCCA Protein Family

**DOI:** 10.3390/plants9091092

**Published:** 2020-08-25

**Authors:** Igor I. Turnaev, Konstantin V. Gunbin, Valentin V. Suslov, Ilya R. Akberdin, Nikolay A. Kolchanov, Dmitry A. Afonnikov

**Affiliations:** 1 Institute of Cytology and Genetics, SB RAS, 630090 Novosibirsk, Russia; turn@bionet.nsc.ru (I.I.T.); genkvg@bionet.nsc.ru (K.V.G.); valya@bionet.nsc.ru (V.V.S.); akberdinir@gmail.com (I.R.A.); kol@bionet.nsc.ru (N.A.K.); 2 Biosoft.ru, 630058 Novosibirsk, Russia; 3 Faculty of Natural Sciences, Novosibirsk State University, 630090 Novosibirsk, Russia; 4 Kurchatov Genomics Center, Institute of Cytology and Genetics, SB RAS, 630090 Novosibirsk, Russia

**Keywords:** auxin biosynthesis, YUCCA, flavin-dependent monooxygenase, phylogeny, charophytes, horizontal gene transfer

## Abstract

YUCCA (YUCCA flavin-dependent monooxygenase) is one of the two enzymes of the main auxin biosynthesis pathway (tryptophan aminotransferase enzyme (TAA)/YUCCA) in land plants. The evolutionary origin of the YUCCA family is currently controversial: YUCCAs are assumed to have emerged via a horizontal gene transfer (HGT) from bacteria to the most recent common ancestor (MRCA) of land plants or to have inherited it from their ancestor, the charophyte algae. To refine YUCCA origin, we performed a phylogenetic analysis of the class B flavoprotein monooxygenases and comparative analysis of the sequences belonging to different families of this protein class. We distinguished a new protein family, named type IIb flavin-containing monooxygenases (FMOs), which comprises homologs of YUCCA from Rhodophyta, Chlorophyta, and Charophyta, land plant proteins, and FMO-E, -F, and -G of the bacterium *Rhodococcus jostii* RHA1. The type IIb FMOs differ considerably in the sites and domain composition from the other families of class B flavoprotein monooxygenases, YUCCAs included. The phylogenetic analysis also demonstrated that the type IIb FMO clade is not a sibling clade of YUCCAs. We have also identified the bacterial protein group named YUC-like FMOs as the closest to YUCCA homologs. Our results support the hypothesis of the emergence of YUCCA via HGT from bacteria to MRCA of land plants.

## 1. Introduction

YUCCA (YUCCA flavin-dependent monooxygenase) in higher plants is an important enzyme of the biosynthesis of auxin (indole acetic acid (IAA)), a hormone involved in the regulation of all main processes of plant growth and differentiation [1,2]. This hormone is necessary for regular embryogenesis, shoot growth, development of the root, hypocotyl, the lateral organs of the aboveground part of plants, differentiation of the vascular system cells, phyllotaxis, gravitropism [3,4,5], and stress response [6,7]. Elevated IAA levels or enhanced auxin signaling can promote disease development in some plant–pathogen interactions and antagonize plant defense responses [8]. Plants utilize auxin signaling and transport to modify their root system architecture when responding to diverse biotic and abiotic rhizosphere signals [9,10]. It is no wonder that its biosynthesis, regulation, and metabolism in plants and evolution of the involved genes attract so much attention of researchers [11,12,13,14,15].

In land plants, YUCCAs provide the second stage of the indole-3-pyruvate (IPA) pathway of auxin biosynthesis from tryptophan in a two-stage pathway; the first reaction is performed by the tryptophan aminotransferase enzyme (TAA) [16,17,18,19,20,21,22]. In this way, the canonical pathway of auxin biosynthesis can be realized only when both functional enzymes, TAA and YUCCA, are coded in the genome. Thus, the evolution of the TAA and YUCCA proteins is tightly related to the origin of the auxin biosynthesis pathway in plants, which is still controversial.

Yue et al. [23] found no homologs of the TAA and YUCCA proteins in the green algae species. Therefore, they suggested that these two enzymes originated in the the most recent common ancestor (MRCA) of the land plants by horizontal gene transfer (HGT) from non-plant species. However, research on the genome of the charophyte alga *Klebsormidium nitens* NIES-2285 (syn. *K. flaccidum* NIES-2285; *K. flaccidum* NIES-2285 was reidentified as *K. nitens* NIES-2285, June 2016 [24]) by Wang et al. [25] identified both TAA (kfl00051_0080) and YUCCA (kfl00109_0340; NCBI ID GAQ82387.1) homologs. This fact allowed the authors to hypothesize about the early emergence of the auxin biosynthesis pathway in Charophytes [25], which are the ancestors of land plants, i.e., before the plants started to colonize the land [26]. The debate on the presence of a functional TAA enzyme in charophyte algae and, correspondingly, the existence of the functional IPA auxin biosynthesis pathway in this taxon persist in the scientific literature [27,28,29,30]. The evolution and function of YUCCA proteins, however, have not received detailed consideration [30].

YUCCA proteins belong to the flavoprotein monooxygenases superfamily [31]. The flavoprotein monooxygenases are involved in a wide range of biological processes of living organisms, from catabolism, detoxification, and biosynthesis to the emission of light and control of axons. They catalyze the incorporation of one molecular oxygen atom into the substrate, while the second oxygen atom is reduced to water [32]. The superfamily of flavoprotein monooxygenases has been found in animals, plants, fungi, bacteria, and archaea [33,34,35]. The superfamily is subdivided according to the protein structure and enzyme properties into eight classes, A–H [32,36]. The class of B flavoprotein monooxygenases comprises three subclasses: *N*-hydroxylating monooxygenases (NMOs), Baeyer–Villiger monooxygenases (BVMOs), and flavin-containing monooxygenases (FMOs) [37]. The YUCCA family belongs to FMOs [31,38].

All class B flavoprotein monooxygenases are able to oxidize both carbon atoms and other heteroatoms [36]. In addition, all class B flavoprotein monooxygenases contain two typical Rossmann fold motifs (GxGxxG/A). The first motif, the FAD binding site, is located closer to the N end and the second, the NADPH binding site, to the central part of the protein sequence [37,39,40]. An FMO characteristic sequence motif (FxGxxxHxxxF/Y/W) resides between them near the second Rossmann fold motif. It is noted that the last amino acid of the motif in the FMO subclass protein sequences is F/Y, and, in the BVMO subclass, it is always W [37].

The exception of all class B flavoprotein monooxygenases is only the subclass of NMO proteins, which carries a single conserved histidine (xxxxxxHxxxx) in the region of the FMO motif. NMOs usually mediate the FAD-dependent oxidation of primary amines with a long chain [41,42]. The BVMO proteins first and foremost catalyze Baeyer–Villiger oxidation, but they are also able to oxidize heteroatom-containing compounds (the compounds containing N, S, B, or Se). On the contrary, FMOs specialize in the oxidation of heteroatom-containing compounds and are ineffective as catalysts for Baeyer–Villiger oxidation [42]. FMOs are involved in several oxidative biological processes, drug detoxification, and biodegradation of aromatic compounds [43]. That is why the animal FMOs were first studied as the enzymes capable of degrading xenobiotics and thus assisting the body to eliminate toxic compounds [42,44,45]. Only recently have FMOs also been considered as biocatalysts, thanks to the identification and manufacture of bacterial FMOs, which, unlike the animal homologs, are easily isolatable as soluble proteins [46].

Here, the phylogeny of the B flavoprotein monooxygenases is studied in detail, which suggests that the type II FMO group comprises three subgroups: IIa, IIb, and IIc. Based on these data, the origin and evolution of proteins of the YUCCA family are assumed.

## 2. Results

### 2.1. Analysis of the Proteins of Class B Flavoprotein Monooxygenases

**Figure 1:**
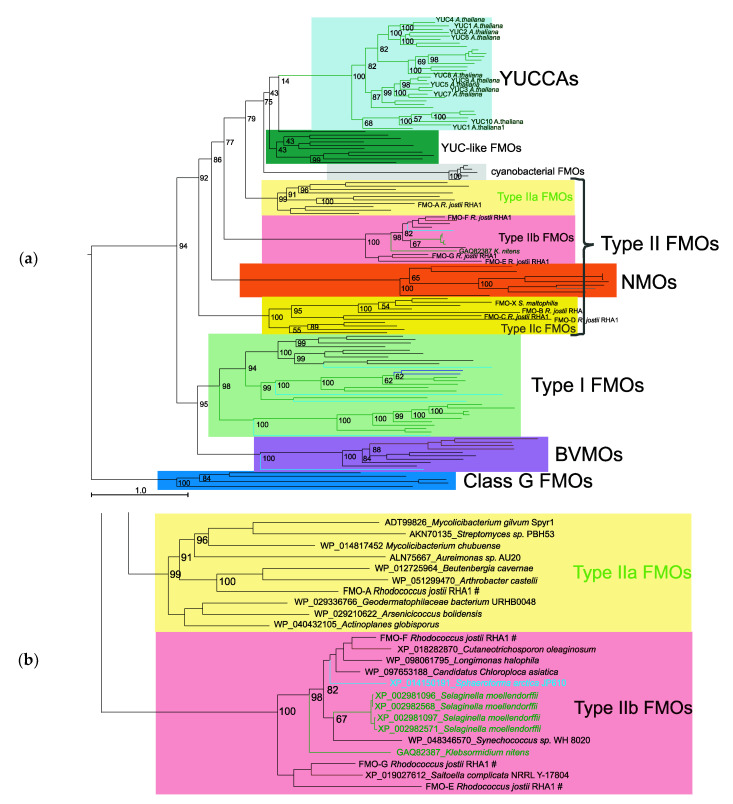
Phylogeny of the class B flavoprotein monooxygenases reconstructed using IQ-TREE. The branches of green alga and land plant proteins are green; of fungi, brown; of protists, cyan; of animals, blue; of bacteria, black; of archaebacteria, gray. (**a**) Phylogenetic tree of class B flavoprotein monooxygenases with the class G flavoprotein monooxygenases as an outgroup (dark blue background). (**b**) A fragment of the phylogenetic tree of class B flavoprotein monooxygenases comprising two groups of proteins: type IIa flavin-containing monooxygenases (FMOs) and type IIb FMOs; # denotes protein sequences from Riebel et al. [40]; the remaining designations are as in (**a**). The numbers near the branches represent bootstrap support values.

In order to clarify the evolutionary origin of the YUCCA family proteins, we performed a phylogenetic analysis of class B flavoprotein monooxygenases. For this purpose, the homologs of YUCCA proteins were searched in the NCBI amino acid sequence database using BLASTP with e-value = 1 × 10^–5^ (see Materials and Methods). The sample was supplemented with the proteins from Riebel et al. [40], namely, seven amino acid sequences of FMO-A to FMO-G proteins of the bacterium *Rhodococcus jostii* RHA1 and the FMO-X of the bacterium *Stenotrophomonas maltophilia*. Riebel et al. [40] performed phylogenetic and experimental analyses of these proteins and proposed that they fall into separate clusters on the phylogenetic tree of the B flavoprotein monooxygenases, termed as type II FMOs [40]. A phylogenetic tree was constructed for the sequences of the class B flavoprotein monooxygenases (class_B_FMO_proteins sequence set; Supplementary Data File 1: class-B-FMO-134-prot-aln.fasta) with the help of the IQ-TREE program (Figure 1a). Class G flavoprotein monooxygenase proteins were used as an outgroup.

The phylogenetic tree (Figure 1a) allowed us to distinguish the proteins belonging to three subclasses of the class B flavoprotein monooxygenases. The first group of FMO subclass proteins, YUCCAs, is well separated and comprises only plant proteins (Figure 1a, pale blue background); the second group, YUC-like FMOs, is represented only in bacteria (green background); the third, cyanobacterial FMOs, is found only in cyanobacteria, with a long branch leading to it (gray background). The fourth group of proteins, type II FMOs (pale yellow, pink, and bright yellow) appears to be the most heterogeneous. This group, containing bacterial, plant, fungal, and protist proteins, splits into three subgroups, which we named type IIa FMOs (Figure 1a, pale yellow background), type IIb FMOs (pink background), and type IIc FMOs (bright yellow background). The sequences of type IIa FMOs and type IIc FMOs are observable only in bacteria, whereas the type IIb FMO subgroup also contains plant, fungal, and protist sequences in addition to the bacterial ones (Figure 1b). The last group of proteins, type I FMOs (Figure 1a, pale green background), is represented by bacterial, protist, plant, and animal proteins, which have mainly been well studied [40]. The second subclass of the class B flavoprotein monooxygenases, NMOs (Figure 1a, orange background), is represented by bacterial and fungal proteins, and the third subclass, BVMOs (Figure 1a, violet background), by fungal, protist, and bacterial ones.

The type IIb FMO clade also comprises the sequences of plant organisms: four sequences of the lycophyte *Selaginella moellendorffii* and the YUCCA homolog from the charophyte alga *K. nitens*, GAQ82387.1 [25].

The sequences of *R. jostii* RHA1 FMO-E, FMO-G, and FMO-F proteins [40] also belong to the type IIb FMOs, while the *R. jostii* RHA1 FMO-A protein falls into the type IIa FMO clade. Other *R. jostii* RHA1 proteins from the same study (FMO-B, FMO-C, and FMO-D), as well as the *S. maltophilia* FMO-X, belong to type IIc FMOs (Figure 1a).

To estimate the robustness of the tree reconstruction of B flavoprotein monooxygenase proteins, we additionally used RaxML (Supplementary Data File 2, Figure S1a) and mrBayes programs (Supplementary Data File 2, Figure S1b).

The cladograms of the three trees for class B flavoprotein monooxygenases were constructed using IQ-TREE, and these programs are shown in Figure S3a (Supplementary Data File 2). The topology of the RAxML tree is similar to that of the IQ-TREE for these proteins. The small difference is the change in positions of the cyanobacterial FMO and YUC-like FMO clades: in the IQ-TREE, the YUC-like FMO clade is the closest to the YUCCAs, followed by cyanobacterial FMOs, versus the opposite positions in the corresponding RAxML tree. The topology of class B flavoprotein monooxygenase proteins constructed using mrBayes differs from those of the IQ-TREE and RAxML trees remarkably. In the mrBayes tree, the cyanobacterial FMO and type IIb FMO clades are the closest to the YUCCA clade (Figure S3a, Supplementary Data File 2). In addition, the mrBayes phylogeny for the class B flavoprotein monooxygenase proteins is only partially resolved: basal tetrafurcation in the tree with all major clades of class B flavoprotein monooxygenases (red line in the mrBayes cladogram; Figure S3a, Supplementary Data File 2) is observed.

### 2.2. Comparative Analysis of the Functional Sites and Domains of Class B Flavoprotein Monooxygenases

**Figure 2:**
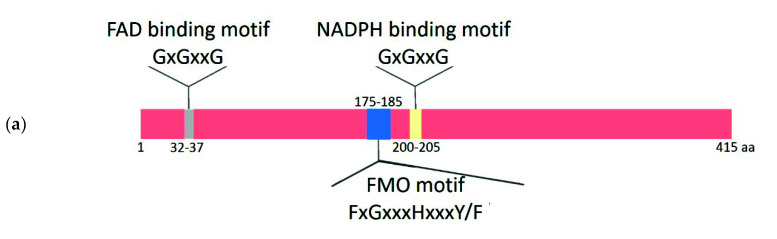

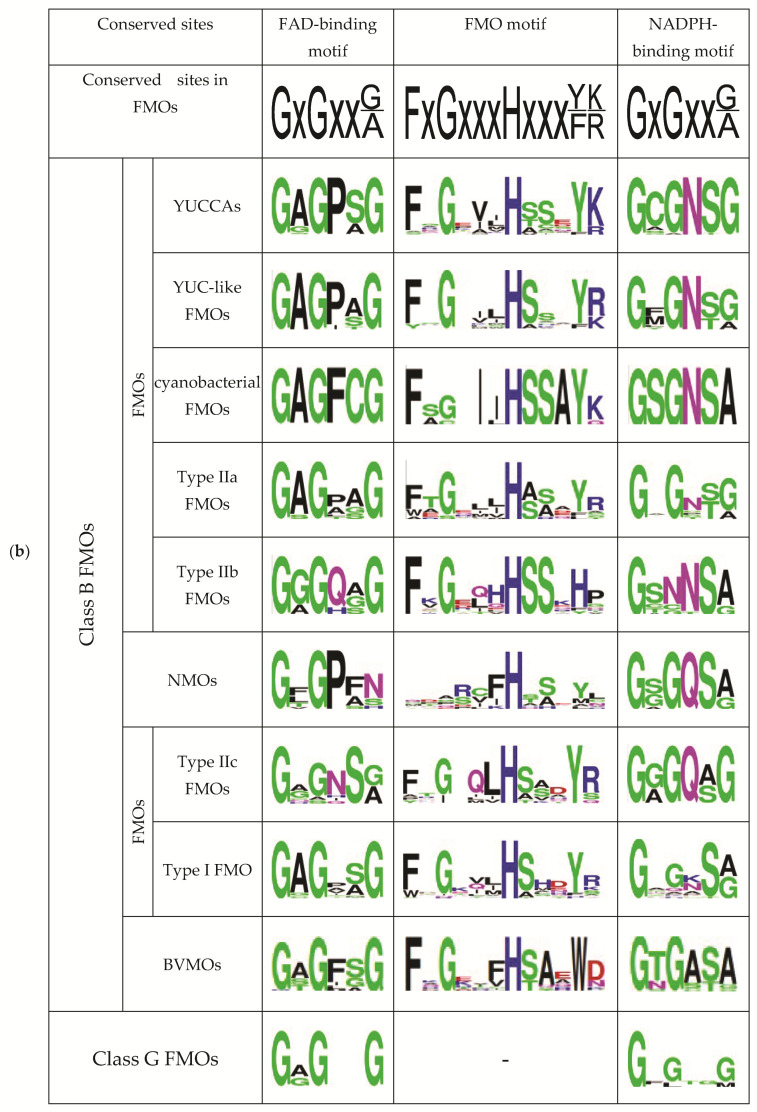
WebLogo representation of the key functional sites in the FMO/ Baeyer–Villiger monooxygenase (BVMO) protein homologs. (**a**) Scheme of arrangement of the conserved motifs in FMO proteins (the amino acid positions are shown according to the *A. thaliana* YUC2 sequence, AT4G13260). (**b**) The consensus of the three motifs—FMO-binding, FMO, and nicotinamide adenine dinucleotide (NADH)-binding (Columns 2–4, respectively).

We performed a comparative analysis of the protein sequences for the three functional motifs (Figure 2a) in all ten groups of proteins that we distinguished in the B flavoprotein monooxygenase phylogenetic tree, namely, YUCCAs, YUC-like FMOs, cyanobacterial FMOs, type IIa FMOs, type IIb FMOs, type IIc FMOs, NMOs, type I FMOs, BVMOs, and class G flavoprotein monooxygenases (Figure 1a). Figure 2a shows the arrangement of FAD-binding, FMO, and NADPH-binding motifs in an FMO sequence, *A. thaliana* YUC2 AT4G13260 [38], and, in Figure 2b, the motifs in a WebLogo format [47] for the ten groups of sequences.

The considered groups appeared to be homogeneous in the sequences of the motifs they carried except for NMOs and class G flavoprotein monooxygenases (Figure 2b). The former carries a single conserved histidine in the region of the FMO motif (xxxxxxHxxxx), while the latter does not have an FMO motif at all (Figure 2b). As evident from Figure 2b (Column 2), the FAD-binding motif in the sequences of all protein groups, except for the type IIc FMOs and NMOs, is similar and contains three highly conserved glycines. The third glycine in type IIc FMOs is frequently replaced with alanine (A) versus NMOs, where the third glycine, in most cases, is replaced with asparagines (N). The FxGxxxHxxxY/FK/R consensus is characteristic of the FMO motif (Figure 2b) in the YUCCA, YUC-like FMO, cyanobacterial FMO, type IIa FMO, type IIc FMO, and type I FMO groups, with the prevalence of tyrosine (Y) at the next-to-last position. The next-to-last symbol in this motif for BVMOs is a conserved tryptophan (W). As for type IIb FMO sequences, the FxGxxxHxxx(H/y/f)P consensus is characteristic of them. The next-to-last symbol of this motif is a conserved histidine (H) in 70% of the sequences and Y or F in the remaining 30% of sequences; the latter variant (y/f) is characteristic of the other FMO groups (type I FMOs, type IIa FMOs, type IIc FMOs, cyanobacterial FMOs, YUC-like FMOs, and YUCCAs). However, histidine in this position in all FMO groups, except for type IIb FMOs, is observable only twice in the type I FMO cluster. Proline (P) is present at the last position of this motif in type IIb FMOs versus either lysine (K) or arginine (R) in the remaining FMO groups.

As for the NADPH-binding motif, all three conserved glycines are characteristic of the YUCCAs, type IIc FMOs, and class G flavoprotein monooxygenases. On the contrary, the third glycine is frequently replaced with alanine (A) in YUC-like FMOs, type IIa FMOs, type IIb FMOs, NMOs, and type I FMOs. Finally, the characteristic of the cyanobacterial FMOs and BVMOs is a highly conserved alanine at the last position. The type IIb FMO proteins have asparagines (N) instead of the second glycine in 57% of cases; however, this amino acid in the nine remaining groups of proteins is absent in this site. The amino acids of the NADPH-binding motif in the type I FMO group are analogous to those in type IIa FMO, type IIc FMO, cyanobacterial FMO, YUC-like FMO, and YUCCA groups.

Thus, the consensus of the FMO and NADPH-binding motifs of most proteins belonging to the type IIb FMO clade contains the amino acids atypical of type IIa and type IIc FMO proteins. It can be noted that the FMO motif of the *K. nitens* GAQ82387.1 protein, belonging to type IIb FMOs, contains H amino acid at the next-to-last position (the last is glycine, G), which is also observable at this position in the *R. jostii* RHA1 FMO-F protein [40].

**Figure 3:**
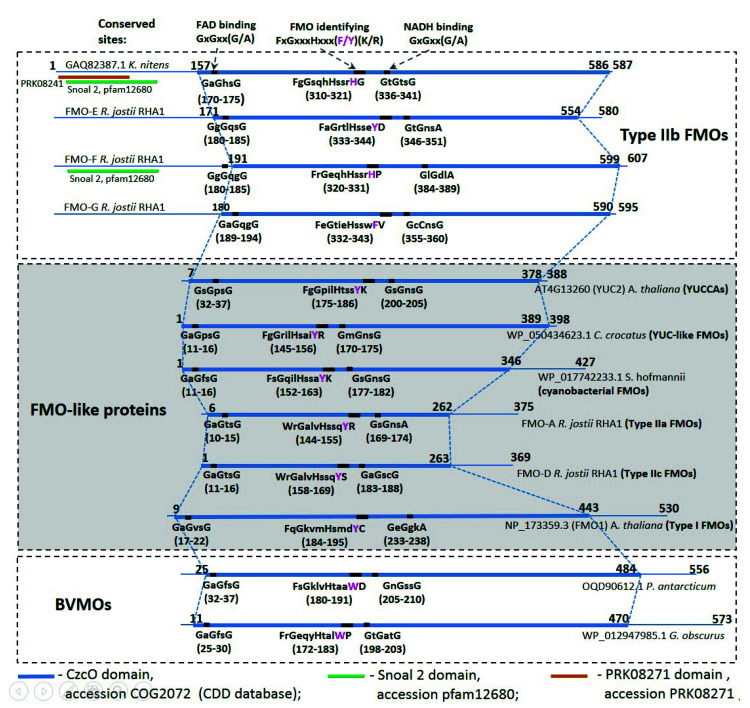
A comparison of the conserved domains of three groups of proteins: type IIb FMOs, FMO-like (YUCCA flavin-containing monooxygenase (YUCCAs), YUC-like FMOs, cyanobacterial FMOs, type IIa FMOs, type IIc FMOs, and type I FMOs), and BVMOs. Blue, green, and brown bold lines show the domains in the protein sequences from CDD (Conserved Domains Database) v. 3.18. Abbreviations: *C. crocatus*, bacterium *Chondromyces crocatus*; *S. hofmannii*, cyanobacterium *Scytonema hofmannii*; *P. antarcticum*, fungus *Penicillium antarcticum; G. obscures*, bacterium *Geodermatophilus obscures.*

We performed a comparative analysis of the conserved domains of three groups of proteins, namely, BVMOs, type IIb FMOs, and FMO-like proteins, which comprise type I, type IIa, and type IIc FMOs, as well as cyanobacterial FMOs, YUC-like FMOs, and YUCCAs (Figure 3). The YUCCA, YUC-like FMO, cyanobacterial FMO, type IIa FMO, type IIc FMO, and type I FMO proteins were pooled into one group of FMO-like proteins since they have only insignificant differences in the sequences of the conserved sites examined in this work (Figure 2b). The class G flavoprotein monooxygenases and NMOs have not been considered in this comparative analysis since the composition of their conserved sites differs considerably from the remaining analyzed groups of proteins.

The search for FMO/BVMO proteins (the proteins belonging to the type IIb FMO, FMO-like, and BVMO groups) of conserved domains from the CDD database succeeded in detecting one main domain, CzcO (accession COG2072), in all sequences. This domain resides in the central part of the proteins, occupying 65% to 98% of their length (Figure 3). The program hhsearch identifies the Pfam domain PF00743.19 (FMO-like, e-value < 1 × 10^−30^) in this section of the sequence.

As evident from Figure 3, the type IIb FMO sequences have the N-terminal domain with a length of approximately 160 amino acids, which is absent in the remaining type I FMO, type IIa FMO, type IIc FMO, YUC-like FMO, cyanobacterial FMO, and BVMO proteins. This domain is present in all type IIb FMO proteins. The analysis with CD-search software (default e-value threshold 0.01) for individual sequences allowed us to discover domains Snoal2 (SnoaL-like domain; accession pfam12680; e-value = 1.70 × 10^−4^) and RNA polymerase factor sigma−70 (accession PRK08241; e-value = 9.67 × 10^−4^) in this region for the sequence GAQ82387.1 *K. nitens*. For the sequence *R. jostii RHA1* FMO-F, only the SnoaL-like domain (e-value = 4.21 × 10^−3^) has been identified. Both subdomains are located in the region of amino acid residues 3–110 of type IIb FMO proteins (Figure 3). A search using CD-search revealed no similarities of this fragment with known domains for FMO-E and FMO-G sequences.

Analyzing individual sequences with the hhblits program (e-value threshold 0.1) in the Pfam database gave similar results. For FMO-F and GAQ82387.1 sequences, domains in the Pfam PF13577.6 family (SnoaL_4; e-values 0.19 and 0.00061, respectively) have been identified. This type of domain, as well as Snoal2, refers to the superfamily NTF2-like. For FMO-E and FMO-G sequences, known domains have not been identified. To clarify the function of this fragment, we used the search for known Pfam domains in the multiple alignments of type IIb FMO proteins using the program hhsearch. The highest coverage (positions 35–112) was found for domains PF02982.14 (Scytalone_dh; e-value = 0.00041) and PF02136.20 (NTF2; e-value = 0.00049). All these domains, like Snoal2 and SnoaL_4, belong to the NTF2-like domain superfamily.

Thus, the type IIb FMO sequences differ from the FMO-like sequences by the presence of an additional domain at their N end, which probably belongs to the NTF2-like superfamily.

### 2.3. Abundance of the Sequences Homologous to Type IIb FMOs in the Main Taxa

In order to better understand the abundance of the proteins belonging to the type IIb FMO, FMO-like (YUCCAs, YUC-like FMOs, cyanobacterial FMOs, type IIa FMOs, type IIc FMOs, and type I FMOs), and BVMO groups in the main taxa, we did a search for the homologs of the above-listed three groups among the main prokaryotic and eukaryotic taxa. For this purpose, we searched the NCBI database with the help of PHI-BLAST at e-value = 1 × 10^−10^, taking into account the consensus of the FMO motif. The FMO-like proteins were pooled into one group for the PHI-BLAST search since they are indistinguishable according to the consensus of the FMO motif, which is used in this search. The representative protein sequences are taken as a query for type IIb FMOs, FMO-like proteins, and BVMOs listed in Section 4.3. In the PHI-BLAST search, the following consensus of the FMO motif was specified for each of the three examined groups of proteins: (i) type IIb FMOs, FxGxxxHxxxH; (ii) FMO-like proteins (comprising type I FMOs, type IIa FMOs, type IIc FMOs, and YUCCAs, which are indistinguishable from one another in the PHI-BLAST search), FxGxxxHxxxY/F; (iii) BVMOs, FxGxxxHxxxW.

The abundance of the found homologs in the main taxa, taking into account the degree of their similarity, is listed in Table 1.

As evident from Table 1, homologs of type IIb FMO proteins are widely abundant among bacteria and fungi but almost absent in plants and undetectable in animals and archaea. The FMO-like protein homologs appear to be widely abundant in all studied taxa except for archaea. The degree of similarity between the sequences taken as queries in the search for homologs and their fungal and bacterial homologs are considerably higher among type IIb FMOs and BVMOs compared with the FMO-like proteins of different taxa. This is suggested by the fact that a considerable number of close homologs (e-value = 0 to 10^−70^) between bacterial and fungal proteins in our study was found only for the type IIb FMO and BVMO groups rather than for the FMO-like group (Table 1).

### 2.4. Analysis of the Plant Class B Flavoprotein Monooxygenases Represented in Transcriptome Projects

**Figure 4:**
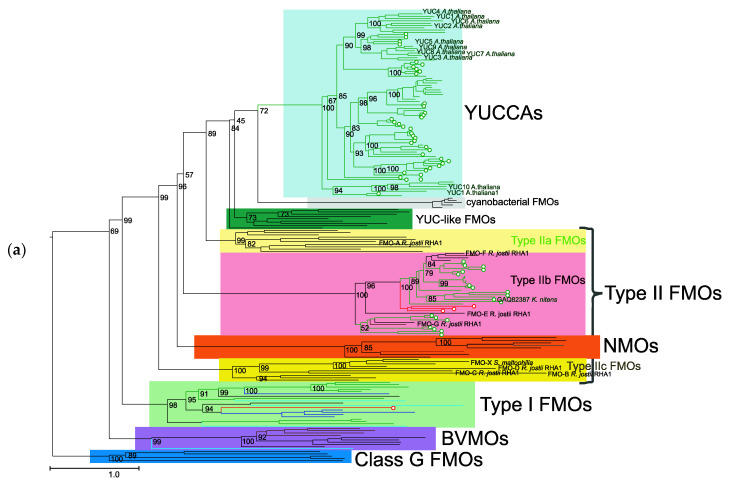

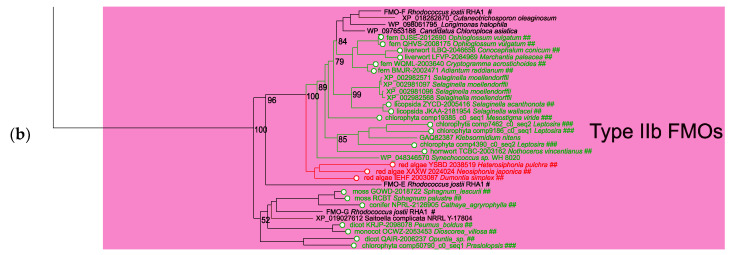
The phylogenetic tree (IQ-TREE method) of class B flavoprotein monooxygenases, including sequences from the transcriptomic assemblies. (**a**) The phylogenetic tree class_B_FMO_proteins_and_transcriptomic, with class G flavoprotein monooxygenases as an outgroup. The sequences from the 1KP project [49] and the Green Algal Tree of Life Project [50] transcriptomic assemblies are marked with a circle. The sequences of green algae and land plants are colored green; of red algae, red; of fungi, brown; of protists, cyan; of animals, blue; of bacteria, black; of archaebacteria, gray. (**b**) A fragment of the phylogenetic tree of FMOs (extended by transcriptome sequences) for the type IIb FMO clade. The designations are the same as in (**a**). Additionally, three protein sequences marked with # are extracted from Riebel et al. [40], with ##, from the 1KP project [49], and with ###, from the Green Algal Tree of Life Project [50]. The numbers near the branches represent bootstrap support values.

In order to better identify FMO sequences in plant organisms, we extended the FMO proteins with homologous sequences from the 1KP [48,49] and The Green Algal Tree of Life [50] transcriptome projects (Supplementary Data File 1, class-B-FMO-195-prot-ext-aln.fasta). The phylogenetic tree of class B flavoprotein monooxygenases extended a sequence set, as shown in Figure 4a. The tree contains all main protein groups of class B flavoprotein monooxygenases that we distinguished in Figure 1, as well as the class G flavoprotein monooxygenase proteins as an outgroup. The clade of type IIb FMO proteins is shown in more detail in Figure 4b. In addition to the bacterial and fungal proteins, the proteins of red algae (Rhodophyta), green algae (Chlorophyta), charophytes (Charophyta: family Klebsormidiaceae), as well as the main land-plant taxa (mosses, liverworts, hornworts, clubmosses, ferns, conifers, and angiosperms, both monocots and eudicots) are represented. It is noted that the ancestors of the extant land plants and the algae of the Charophyta division, containing the family Klebsormidiaceae, are tightly related [51,52,53].

In order to assess the robustness of phylogeny of the B flavoprotein monooxygenase extended sequence set, we additionally estimated the phylogenetic tree using RAxML (Supplementary Data File 2, Figure S2a) and mrBayes (Supplementary Data File 2, Figure S2b). These data show that the topologies of the trees obtained using IQ-TREE and RaxML do not differ from one another. The tree estimated by mrBayes (Supplementary Data File 2, Figure S3b) has actually only one difference in the positions of clades relative to the IQ-TREE and RAxML trees, namely, the cyanobacterial FMO and YUC-like FMO clades change their positions so that the YUC-like FMOs become the closest to YUCCAs, followed by cyanobacterial FMOs. The mrBayes tree is also underresolved since it contains a basal trifurcation between NMOs, type IIb FMOs, and type IIa FMOs–YUCCAs, denoted with a red line in the mrBayes cladogram.

The results of the identification of the homologs of *K. nitens* GAQ82387.1 and *A. thaliana* YUC2 AT4G13260 in the 1KP [49] and NCBI databases are listed in Table 2.

According to the 1KP database (Table 2), the abundance of the homologs of YUCCA (AT4G13260 used as query) and GAQ82387.1 in the angiosperm taxa are drastically different. In this database, the number of YUCCA homologs exceeds 300 among the dicots and is over 40 among monocots versus single homologs of GAQ82387.1, taking into account that the number of analyzed genomes is almost 600 for dicots and over 100 for monocots.

The second interesting result is that the homologs of *K. nitens* GAQ82387.1 are detected in individual algal genomes, namely, in four genomes of lower green algae (Chlorophyta), in red algae, and Streptophyta algae (only in the family Klebsormidiophyceae). However, YUCCA homologs are undetectable in the algae in both the 1KP and NCBI databases.

A relatively high abundance of homologs of both YUCCAs (AT4G13260) and GAQ82387.1 is observed in one of the two fern taxa, Leptosporangiate monilophytes (Table 2): the homologs are present in 19 and 33 representatives of 65, respectively (1KP database).

A high abundance of homologs of both genes in ferns and lower land plants raises the question of whether the homologs of these two genes are simultaneously present in the genome of the same species. We have examined this issue and show the results in Table 3. This table lists the species (Column 1) where the homologs of both GAQ82387.1 and YUCCAs have been identified.

As evident from Table 3, the homologs of YUCCAs and GAQ82387.1 (according to the 1KP database) are simultaneously presented in three liverwort species, two hornwort species, 13 leptosporangiate monilophytes, one monocot species, and two eudicot species.

## 3. Discussion

### 3.1. Type IIb FMOs Is a Novel Family of Class B Flavoprotein Monooxygenases

The reconstruction of the B flavoprotein monooxygenase phylogenetic tree demonstrated that type IIb FMOs (Figure 1 and Figure 4) are distinguished from the other type II FMO sequences (which we refer to the type IIa FMOs and type IIc FMOs). The type IIb FMO cluster is well separated from other groups of B flavoprotein monooxygenases, as shown by different tree reconstruction programs (IQ-TREE, RAxML, and mrBayes) for the two sets of proteins (Supplementary Data File 2; Figures S1a,b and S2a,b). However, its position in the B flavoprotein monooxygenase tree varies depending on the tree reconstruction method and sequence dataset. One possible reason is the influence of the three long branches leading to cyanobacterial FMO, NMO, and type IIb FMO clades, which could introduce bias in the phylogeny reconstruction due to the long branch attraction effect [54]. On the other hand, for some clades (YUC-like bacterial proteins, for instance), the support values of the branches are quite low under both maximum likelihood (IQ-TREE and RAxML) and mrBayes methods. For instance, there is low support to conclude that typeIIb FMOs and cyanobacterial FMOs cluster together in the tree obtained by mrBayes, although strong support is obtained for typeIIb FMOs, regardless of their position in the tree (and inference method). We may conclude, therefore, that this clade is well-defined, but its position in the tree is not well-defined in some of our analyses.

It should be noted, however, that in all the trees obtained, type IIb FMOs are not the closest clade to the YUCCA protein. These are either YUC-like bacterial proteins or cyanobacterial FMOs. Interestingly, the cluster that includes type IIb FMO proteins was identified by Bowman et al. [55] in the search for YUCCA homologs in the *Marchantia polymorpha* genome. Two proteins from *M. polymorpha* were identified in this cluster.

It is important to note that the separate cluster of class B flavoprotein monooxygenases within type II FMOs was earlier identified by Riebel et al. [40]. They analyzed the phylogeny of the FMO proteins and found a new group of type II FMOs, which comprised the sequences from FMO-A to FMO-G of bacterium *R. jostii* RHA1. Correspondingly, they attributed the earlier known and well-studied plant, animal, and bacterial FMOs to type I FMOs. In addition, three of the eight type II FMO proteins in *R. jostii* RHA1, FMO-E, FMO-F, and FMO-G fall into the separate cluster on the type II FMO subtree. These proteins appear to possess an ability, unique for FMOs, to catalyze both sulfoxidation (an ability characteristic of FMOs and BVMOs) and Baeyer–Villiger oxidation (an ability characteristic of BVMOs but not FMOs) [40]. Riebel et al. showed that the biocatalytic activity of the E, F, and G FMOs are more similar to BVMOs than the remaining FMO proteins. In addition, FMO-E, FMO-F, and FMO-G utilize either NADH or NADPH as a cofactor. On the contrary, the remaining FMO proteins from *R. jostii* RHA1 (type I FMOs and type II FMOs, in particular, FMO-A, FMO-B, FMO-C, and FMO-D) typically utilize NADPH as a cofactor [42]. It should also be noted that the *R. jostii* RHA1 FMO-E, FMO-F, and FMO-G proteins have an N-terminal domain with a length of approximately 160 amino acid residues, which are absent in the other earlier-studied class B flavoprotein monooxygenases.

Here, we extended the FMO-E, -F, -G clusters by including sequences from other species. The data on the specific structural features of these proteins and functional motifs and, most importantly, the experimental data of Riebel et al. [40,42] suggest that the proteins of the type IIb FMO clade are a new protein family that differs in structure and function from type IIa FMO and type IIc FMO proteins.

We analyzed the similarity of the N-terminal domain, which is typical for the sequences of this cluster, with known domains in the CDD and Pfam databases. It turned out that these regions may differ from one sequence to another so that for some of them, an individual search does not produce meaningful results, while for others, a significant similarity is detected. However, multiple alignment analysis has shown that these fragments have a remote similarity to NTF2-like superfamily domains. The NTF2-like superfamily is a versatile group of protein domains sharing a common fold [56]. The NTF2-like proteins can be broadly defined into two functional categories: enzymatically active (SnoaL polyketide cyclase, scytalone dehydratase, among others) and enzymatically inactive (ligand-binding) proteins. A low similarity of type IIb FMO sequences with known domains of this superfamily does not allow us, however, to judge their possible function with certainty.

### 3.2. Different Functions of Type IIb FMO and YUCCA Proteins

Our data suggest that the enzymatic functions of type IIb FMO and YUCCA proteins differ. The YUCCA sequences carry a set of three characteristic motifs (Figure 2) and the lack of the N-terminal domain of 160 amino acids, characteristic of type IIb FMO proteins (Figure 3). The taxonomic abundance of YUCCA homologs also differs considerably from that observed for type IIb FMO proteins: they are ever-present in higher land plants according to both the NCBI and 1KP databases [49] versus type IIb FMO proteins, which are detectable in all major taxa except for animals (Table 1 and Table 2). These results are supported by positions of the FMO A-G protein sequences from *R. jostii* RHA1 in the B flavoprotein monooxygenase phylogenetic tree. Three proteins with specific enzymatic properties, FMO-E, -F, -G, cluster with *K. nitens* GAQ82387.1. They have common domain architecture and sequences of the FAD-binding, FMO, and NADH-binding motifs.

We have also shown that both the type IIb FMO and YUCCA proteins are simultaneously present in several liverwort, hornwort, leptosporangiate monilophyte, monocot, and eudicot species (Table 3). This is in agreement with the results of *M. polymorpha* genome analysis [55], indicating the existence of both type IIb FMO and YUCCA homologs in this genome. This implies that these two protein families, in the corresponding plants, serve different functions.

On the other hand, the YUC-like FMO proteins, represented in bacteria (Betaproteobacteria, Deltaproteobacteria, and Bacteroides), appeared to be the closest to YUCCAs in the constructed phylogenetic tree (Figure 1a). These results favor the hypothesis by Yue et al. [23] that YUCCA proteins originated in MRCA of land plants by HGT from bacteria.

### 3.3. The Origin of the Main Auxin Biosynthesis Pathway in Higher Plants

The IPA (indole-3-pyruvate) pathway of auxin biosynthesis involves two enzymes, TAA and YUCCA, which work consecutively. The presence of both enzymes in an organism is necessary to identify IPA auxin biosynthesis. Currently, there are two hypotheses on the origin of the canonical land plant auxin biosynthetic pathway in land plants. Yue et al. [23] have shown that close homologs of both TAA and YUCCA are present only in land plants and absent in algae. Yue et al. suggested that YUCCAs had emerged as a result of HGT from bacteria to the most recent common ancestor (MRCA) of land plants. Wang et al. [25] proposed the existence of this pathway in charophyte algae *K. nitens* and its inheritance by the land plants from charophytes.

In our work, we demonstrated by bioinformatics analysis that land plant YUCCA proteins and their homolog in *K. nitens* (GAQ82387.1) differ in domain structure, functional site composition, and evolutionary patterns. This suggests with a high probability that their enzymatic properties are different. However, it is more important that, earlier in Riebel et al. [40,42], the enzymatic differences between proteins of *R. jostii* RHA1 bacteria belonging to the type IIb FMO group (they have the same domain composition and motives for active sites as GAQ82387.1 proteins) and other representatives of type II FMOs (domain composition and motives of active sites are similar to YUCCA) were experimentally showed. This suggests the absence of the functional canonical auxin biosynthetic pathway in *K. nitens* and implies that this pathway is a land plant innovation [23]. Recent projects on the genome sequencing of charophytes *Penium Margaritaceum* (Zygnematales) [57], *Chara braunii* [57,58], and *Nitella* [28] (Charophyceae) support this hypothesis: neither TAA nor YUCCA homologs were identified in these genomes. These data are consistent with experimental results by Ai et al. [59], who demonstrated that Klebsormidium TAA homologs could not restore the wild-type phenotype of taa mutants in Arabidopsis.

It should be noted, however, that several studies have demonstrated that algae are able to synthesize auxin [60,61,62,63,64]. In particular, a comparison of the genome data on unicellular chlorophytes and higher plants [65] has shown that the former carry several orthologs of genes involved in auxin synthesis and transport but demonstrate a low degree of similarity to YUCCA orthologs (except for *Chlorella vulgaris*) in the absence of TAA orthologs. Thus, auxin biosynthesis in chlorophytes still remains putative, and, if this actually takes place, it might follow alternative pathways (less efficient as compared with the IPA pathway of land plants) [23,65].

Although our data suggest that the type IIb FMO proteins serve different functions than YUCCAs, several questions still remain. Does a high similarity of type IIb FMO sequences indicate that all proteins of this clade have the same function or are there several functions? Are the functions of bacterial type IIb FMOs (for example, FMO-E, FMO-F, and FMO-G) and plant type IIb FMOs (for example, GAQ82387.1) close? Are the functions of the YUCCA clade proteins similar to the functions of the plant type IIb FMO proteins, i.e., are the plant or bacterial type IIb FMOs able to transform IPA into auxin? The precise answers to these questions require further comprehensive studies into the biochemical activities of plant type IIb and other FMO proteins [31].

## 4. Materials and Methods

### 4.1. Sampling of Protein and Transcriptome Sequences and Their Alignment

Two class B flavoprotein monooxygenase samples were included in the analysis: (i) the sample of class B flavoprotein monooxygenase proteins, class_B_FMO_proteins (Supplementary Data File 1, class-B-FMO-134-prot.fasta) and (ii) the sample of class B flavoprotein monooxygenases extended by transcriptome sequences, class_B_FMO_proteins_ext (Supplementary Data File 1: class-B-FMO-195-prot-ext.fasta). The latter contained both the protein sequences of the first sample and the transcriptome sequences homologous to class B flavoprotein monooxygenase proteins.

The class_B_FMO_proteins sample was formed based on several subsamples.

Subsample 1: The homologs of *A. thaliana* YUC2 AT4G13260 were used as query sequences against the PLAZA 2.5 database, which comprises the protein sequences of 25 complete plant genome sequences (five green algae, one moss, one club moss, 13 dicots, and five monocots). The BLASTP program of the PLAZA 2.5 database [66] was used for recognition, utilizing the BLOSUM62 matrix, default parameters, and recognition threshold e-value = 1 × 10^−10^.

Subsample 2: The homologs of *A. thaliana* YUC2 AT4G13260 were searched for among the protein sequences of *Picea abies* (Spruce Genome Project [67,68]). The BLASTP program is available at the database website [69] and was used with the default parameters and recognition threshold e-value = 1 × 10^−10^.

Subsample 3: The homologs of *A. thaliana* YUC2 AT4G13260 were searched for among the protein sequences of nonplant taxa in the NCBI database. The BLASTP program was used for recognition, utilizing the BLOSUM62 matrix, default parameters, and recognition threshold e-value = 1 × 10^−10^.

Subsample 4: The homologs of *K. nitens* GAQ82387.1 were searched for among the protein sequences compiled in the NCBI database. The BLASTP program was used for recognition, utilizing the BLOSUM62 matrix, default parameters, and recognition threshold e-value = 1 × 10^−70^.

Subsample 5: Seven protein sequences (FMO-A to FMO-G), as well as *S. maltophilia* FMO-X, were taken from the paper by Riebel et al. [40].

Subsample 6: Five protein sequences of class G flavoprotein monooxygenases—*Nitrincola lacisaponensis* KDE39435.1 [EC 1.13.12.2], *Agrobacterium vitis* OHZ38954.1 [EC 1.13.12.3], *Bacillus mycoides* OSX95564.1 [EC 1.13.12.3], *Ralstonia solanacearum* CUV18971.1 [EC 1.13.12.3], and *Pseudomonas* sp. Q5W9R9.1 [EC 1.13.12.3]. We used these sequences as an outgroup for the class B flavoprotein monooxygenases because B and G classes form a separate clade in the structure-based phylogeny of Group 1 flavin-dependent monooxygenases [70].

These six subsamples were then pooled into one sample of class B flavoprotein monooxygenases to align the sequences, using the Mafft program [71] available at [72,73], utilizing BLOSUM62 and the default parameters. The sequences that were poorly aligned in the region of the CzcO domain (ACCOG2072) were discarded from the alignment. The position of the CzcO domain in some proteins of class B flavoprotein monooxygenases is shown in Figure 3. The rejection procedure resulted in the elimination of less than 2% of the sequences from the initial sample. Then, a phylogenetic tree was constructed using the RAxML program [74] and redundant sequences in the clusters of the phylogenetic tree were removed. In particular, the proteins of the following species were retained in the YUCCA, plant FMO1, and plant FMO2 clades: *Physcomitrella patens* of bryophytes, *Selaginella moellendorffii* of clubmosses, *Picea abies* of conifers, *Orysa sativa* ssp. *indica* of monocots, and *A. thaliana* of dicots. Plant FMO2 proteins were absent in the *O. sativa* ssp. *indica* and *A. thaliana* genomes; in this case, the proteins of *Zea mays* and *Sorghum bicolor* were retained as the representatives of monocots and *Ricinus communis* and *Theobroma cacao* as representatives of dicots. In the remaining clades of the tree, the number of proteins was reduced by discarding similar redundant sequences, for example, the orthologs of related species. The resulting sample was realigned to construct a new phylogenetic tree. Finally, we obtained the working sample, class_B_FMO_proteins (Supplementary Data File 1, class-B-FMO-134-prot.fasta). It is noteworthy that the set of protein clusters and the affiliation of the sequences with clusters in the phylogenetic tree constructed for the final sample of class B flavoprotein monooxygenases did not change in comparison with the phylogenetic tree constructed using the initial sample.

The class_B_FMO_proteins_ext sample was formed in the following way. The protein sequences of the class_B_FMO_proteins sample were supplemented with two subsamples from the transcriptome projects.

Subsample 1: The homologs of *A. thaliana* YUC2 AT4G13260 were searched for among the transcriptome sequences in the 1KP [48,49] and Green Algal Tree of Life Project [50] databases using BLASTP (BLOSUM62, default parameters, and e-value = 1 × 10^−50^).

Subsample 2: The homologs of the *K. nitens* GAQ82387.1 protein were searched for among the transcriptome sequences in the 1KP [48,49] and the Green Algal Tree of Life Project [50] databases using BLASTP (BLOSUM62, default parameters, and e-value = 1 × 10^−70^).

The pooled sample, comprising the protein sequences of the class_B_FMO_proteins sample and the two above-described subsamples, was aligned using the Mafft program [71] and used to construct a RAxML phylogenetic tree. Then, the transcriptome sequences of monocots and dicots belonging to the YUCCA clade were removed from this sample to decrease the redundancy in this clade of the tree because the monocot and dicot YUCCAs are well represented by the protein sequences from the genome projects. The final sample, class_B_FMO_proteins_ext (Supplementary Data File 1, the class-B-FMO-195-prot-ext.fasta), was used in further work. It should be noted that the set of protein clusters and the affiliation of the sequences with clusters in the phylogenetic tree constructed for the sample of class B flavoprotein monooxygenase proteins plus transcriptome sequences (class_B_FMO_proteins_and_ext sample) did not change in comparison with the phylogenetic tree constructed using the initial sample.

The Promals [75], and Mafft version 7 [76] programs were used for multiple alignments of the sequences of these samples (for both, BLOSUM62 matrix and default parameters were used). First, we aligned core sequences by Promals: FMOs without type IIb FMOs and cyanobacterial FMOs (92 and 129 sequences for the NCBI and extended sample, respectively), BVMOs (10 sequences). Then we added the remained sequences to the core alignment by Mafft. The alignments can be found in Supplementary Data File 1 (for the class_B_FMO_proteins sample, class-B-FMO-134-prot-aln.fasta; for the class_B_FMO_proteins_ext sample, class-B-FMO-195-prot-ext-aln.fasta).

### 4.2. Phylogenetic Analysis

The phylogenetic analysis was performed using a maximum likelihood method implemented in IQ-TREE version 1.6.12 [77] and RAxML version 8.2.4 [74]. In the IQ-TREE variant, the free rate LG + F + R6 evolution model was selected automatically for the tree based only on class_B_FMO_proteins sequences and LG + F + R7 for the tree involving class_B_FMO_proteins_ext sequences. In RAxML, the PROTGAMMALGF model was used (the model selection was performed by the ProteinModelSelection.pl script provided on the RAxML website). In addition, the Bayesian method was implemented in the mrBayes program v. 3.2.5 [78]. Three independent runs with 12 chains each were calculated simultaneously for 1,000,000 generations, sampling every 100 generations. The posterior probability values were generated after discarding the first 25% of the sampled trees. We set prior probability distribution for the amino acid model to mixed; WAG was identified as the model with the maximal posterior probability. The proportion of invariable sites model was combined with the gamma model to describe the rate variation across sites. The number of gamma categories was set to 6.

### 4.3. Analysis of Conserved Sites, Protein Domains, and Taxonomic Representation

The consensus of conserved sites in the FMO proteins was determined using the WebLogo v. 2.8.2 [47].

To identify putative domains in protein sequences, we used the CD-search tool on the NCBI website [79]. Additionally, we used the hhblits tool from the HH-suite3 package [80] for searching protein domains from the Pfam database [81]. We searched for protein domains in aligned type IIb sequences using the hhsearch tool [80].

To analyze the abundance of proteins carrying the conserved site characteristic of the FMO-like, type IIb FMOs, and BVMOs homologs of plant *Populus trichocarpa* XP_002312911.2 and bacterial *Actinobacteria bacterium* OK074 WP_054213635.1 proteins (for FMO-like proteins), bacterial *Halomonas lutea* WP_019017022.1 FMO protein (for type IIb FMOs), and *G. obscurus* WP_012947985.1 protein (for BVMOs) were searched for among the prokaryotes and eukaryotes using PHI-BLAST of NCBI. PHI-BLAST detects proteins with a specified degree of homology, provided the desired sequences carry the specified consensus of conserved sites. The used recognition threshold was e-value = 1 × 10^−5^ and the consensus of the FMO-identifying motif, FxGxxxHxxx[Y/F] for FMO-like proteins, FxGxxxHxxxW for BVMOs, and FxGxxxHxxxH for type IIb FMOs.

## 5. Conclusions

Here, the phylogeny of B flavoprotein monooxygenases has been studied in detail, aiming to resolve the relationship between YUCCA and *K. nitens* GAQ82387.1 proteins. We have demonstrated that the group of proteins named type II FMOs by Riebel et al. [40] falls into three clades, which we refer to as the type IIa FMOs, type IIb FMOs, and type IIc FMOs. The type IIb FMO proteins, which also include the *K. nitens* GAQ82387.1 protein and bacteria *R. jostii* RHA1 FMO-E, -F, -G proteins, differ in the amino acid composition of their sites, protein domains, abundance in different taxa, and, probably, their function from YUCCAs.

Phylogenetic analysis has shown that the type IIb FMO clade is not a sibling clade to YUCCA proteins. Our results favor the hypothesis by Yue et al. [23], asserting that YUCCAs had emerged via a horizontal gene transfer from bacteria to the most recent common ancestor of land plants.

## Figures and Tables

**Table 1:** Abundance of the homologs of the three groups of proteins in the main taxa, depending on the degree of their similarity (NCBI database).

Protein Groups	Animal	Fungi	Plants	Bacteria	Archaea
Type IIb FMOs *					
0 to 10^−70^ **	0	419	4	1387	1
10^−70^ to 10^−40^	0	80	0	2	0
10^−40^ to 10^−5^	0	8	3	19	0
All homologs	0	507(285) ***	7(4)	1408(1041)	1(1)
BVMOs					
0 to 10^−70^	8	918	1	3155	1
10^−70^ to 10^−40^	4	3475	2	1090	9
10^−40^ to 10^−5^	4	1198	1	1605	1
All homologs	16(6)	5591(549)	4(2)	5850(1656)	11(9)
FMO-like plant					
0 to 10^−70^	0	0	512	0	0
10^−70^ to 10^−40^	0	0	16	4	0
10^−40^ to 10^−5^	2486	417	302	2052	0
All homologs	2486(409)	417(244)	831(106)	2056(1306)	0
FMO-like bacteria					
0 to 10^−70^	0	0	0	329	0
10^−70^ to 10^−40^	2	0	612	537	0
10^−40^ to 10^−05^	2917	1305	993	8660	0
All homologs	2919(187)	1305(433)	1605(114)	9526(837)	0

* type IIb FMOs: homologs of *H. lutea* WP_019017022.1. BVMOs: homologs of *G. obscurus* WP_012947985.1. FMO-like plant: homologs of *P. trichocarpa* XP_002312911.2. FMO-like bacteria: homologs of the *A. bacterium* OK074 WP_054213635.1 FMO protein. ** E-value intervals for PHI-BLAST hits. “All homologs” implies the number of homologs for the e-value range of 0 to 10^−5^. *** The number of species with recognized homologs is parenthesized.

**Table 2:** The search for the homologs of *K. nitens* GAQ82387.1 (type IIb FMO group) and *A. thaliana* YUC2 AT4G13260 (YUCCA group) in the 1KP and NCBI databases.

Taxa	GAQ82387.1 Homologs in the 1KP Database	GAQ82387.1 Homologs in the NCBI Database	YUCCA Homologs in the 1KP Database	YUCCA Homologs in the NCBI Database
Eudicots (596)	4(3)	-	434(333)	1680(114)
Monocots (104)	1(1)	-	47(35)	444(26)
Conifers (73)	1(1)	-	4(4)	-
Cycadales (4)	-	-	2(2)	-
Leptosporangiate monilophytes (65)	90(33)	-	25(19)	-
Eusporangiate monilophytes (12)	1(1)	-	-	-
Lycophytes (22)	5(4)	6(1)	-	10(1)
Hornworts (9)	9(5)	-	3(3)	-
Liverworts (28)	17(9)	2(1)	10(10)	6(2)
Bryophyta (41)	1(1)	-	24(18)	6(1)
Zygnemophyceae (5)	-	-	-	-
Coleochaetophyceae (3)	-	-	-	-
Charophyceae (1)	-	-	-	-
Mesostigmatophyceae (1)	-	-	-	-
Chlorokybophyceae (1)	-	-	-	-
Kebsormidiophyceae (2)	2(2)	1(1)	-	-
Green algae (152)	5(4)	1(1)	-	-
Red algae (28)	3(3)	-	-	-

Column 1 shows the taxa according to the NCBI classification (the number of species for each taxon is parenthesized) and Columns 2–5, the number of homologous sequences (the number of the species carrying homologs is parenthesized).

**Table 3:** The plant species with detected homologs of both *K. nitens* GAQ82387.1 and *A. thaliana* YUC2 AT4G13260 in the 1KP database.

Species Identifier in the 1000 Plants (1KP) Database	Taxa	Number of GAQ82387.1 Homologs	Number of YUCCA Homologs
TVSH_201823_Bituminaria_bituminosa	Core Eudicots/Fabaceae	1	2
WWQZ_211706_Medinilla_magnifica	Core Eudicots/Myrtiflorae	1	1
OCWZ_200432_Dioscorea_villosa	Monocots/Dioscoreaceae	1	2
AFPO_201018_Blechnum_spicant	Leptosporangiate monilophytes	4	1
BMJR_200209_Adiantum_tenerum	Leptosporangiate monilophytes	2	2
DCDT_207190_Cheilanthes_arizonica	Leptosporangiate monilophytes	3	1
FLTD_200266_Pteris_ensigormis	Leptosporangiate monilophytes	1	2
GANB_201380_Cyathea_spinulosa	Leptosporangiate monilophytes	4	1
KIIX_201108_Pilularia_globulifera	Leptosporangiate monilophytes	2	1
KJZG_200972_Asplenium_platyneuron	Leptosporangiate monilophytes	5	1
NDUV_201591_Vittaria_appalachiana	Leptosporangiate monilophytes	1	2
NOKI_201577_Lindsaea_linearis	Leptosporangiate monilophytes	5	1
PNZO_215202_Culcita_macrocarpa	Leptosporangiate monilophytes	1	1
RICC_200988_Cystopteris_reevesiana	Leptosporangiate monilophytes	3	1
UFJN_208949_Diplazium_wichurae	Leptosporangiate monilophytes	3	1
UOMY_200602_Osmunda_sp.	Leptosporangiate monilophytes	1	1
WQML_200900_Cryptogramma_ acrostichoides	Leptosporangiate monilophytes	1	2
YLJA_207326_Polypodium_amorphum	Leptosporangiate monilophytes	2	1
RXRQ_201835_Phaeoceros_carolinianus	Hornworts	3	1
TCBC_200001_Megaceros_vincentianus	Hornworts	1	1
HMHL_201008_Marchantia_paleacea	Liverworts	2	1
ILBQ_200700_Conocephalum_conicum	Liverworts	2	1
TXVB_207470_Lunularia_cruciata	Liverworts	3	1
RCBT_Sphagnum palustre	Mosses	1	1

## Supplementary Materials

The following are available online at https://www.mdpi.com/2223-7747/9/9/1092/s1. Supplementary file in ZIP format—“ Supplementary file 1.zip”: (1) class_B_FMO_proteins sample in FASTA format, class-B-FMO-134-prot.fasta; (2) class_B_FMO_proteins_ext sample in FASTA format, class-B-FMO-195-prot-ext.fasta; (3) class_B_FMO_proteins alignment in FASTA format, class-B-FMO-134-prot-aln.fasta; (4) class_B_FMO_proteins_ext alignment in FASTA format, class-B-FMO-195-prot-ext-aln.fasta. Supplementary files in PDF format—“Supplementary file 2.pdf”: Phylogenetic trees of class B flavoprotein monooxygenases obtained by RAxML and mrBayes programs.

## Abbreviations

BVMO	Baeyer–Villiger monooxygenases
FAD	Flavin adenine dinucleotide
NADPH	Nicotinamide adenine dinucleotide phosphate
NADH	Nicotinamide adenine dinucleotide
CDD	Conserved Domains Database
1KP	1000 plants
FMO	Flavin-containing monooxygenase
MRCA	Most recent common ancestor
IAA	Indole acetic acid
IPA	Indole-3-pyruvate
NMO	N-hydroxylating monooxygenases
YUCCA	YUCCA flavin-containing monooxygenase
TAA	Tryptophan aminotransferase enzyme
HGT	Horizontal gene transfer
