# Supplementary material for: The Phylogeny of Class B Flavoprotein Monooxygenases and the Origin of the YUCCA Protein Family
Source: Plants (Basel). 2020 Aug 25;9(9):1092. doi: 10.3390/plants9091092 (PMC7570161; doi:10.3390/plants9091092)
Supplement: Supplementary file 1 [file plants-09-01092-s001.zip › Supplementary file 2.pdf]

Supplementary materials for Turnaev et al. 2020

Phylogenetic trees for class B flavoprotein monooxygenases.

(a)

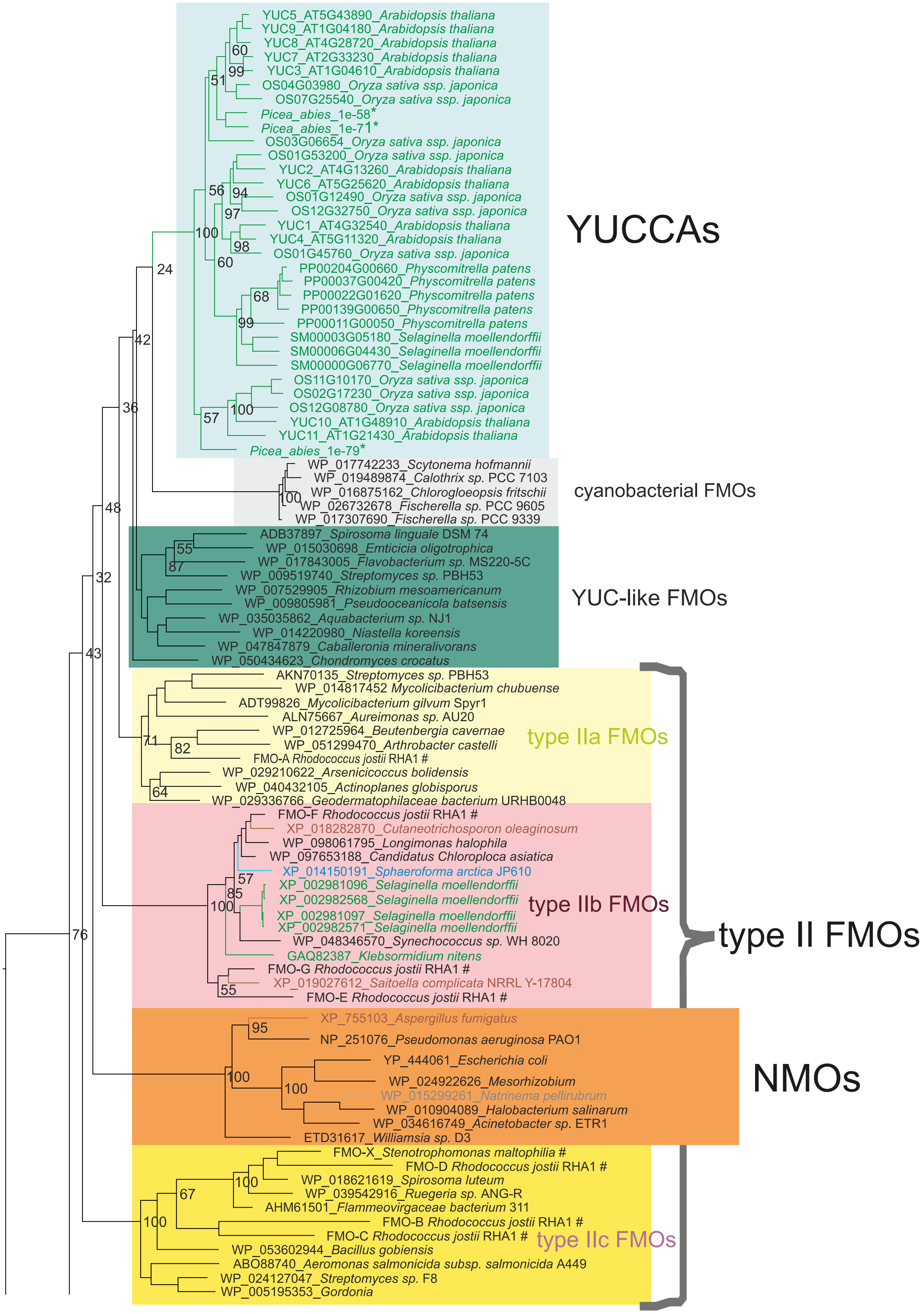

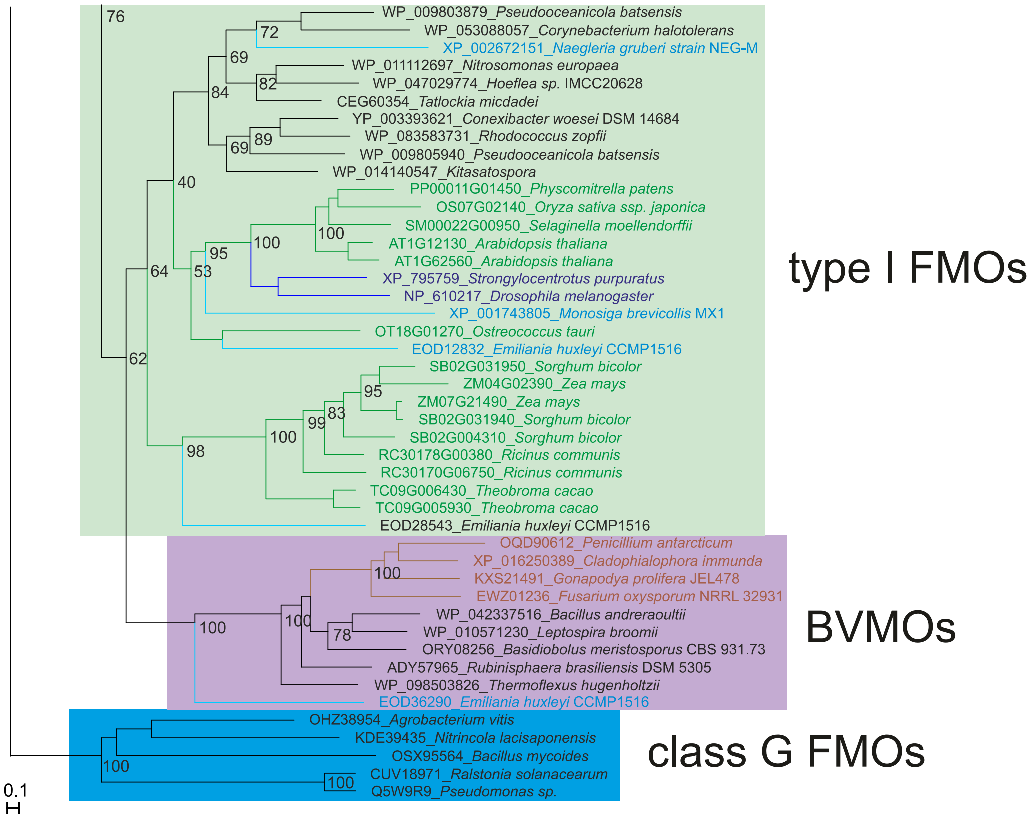
WP_009803879_Pseudooceanicola batsensis
WP_053088057_Corynebacterium halotolerans
XP_002672151_Naegleria gruberi strain NEG-M
WP_011112697_Nitrosomonas europaea
WP_047029774_Hoeflea sp. IMCC20628
CEG60354_Tatlockia micdadei
YP_003393621_Conexibacter woesei DSM 14684
WP_083583731_Rhodococcus zopfii
WP_009805940_Pseudooceanicola batsensis
WP_014140547_Kitasatospora
PP00011G01450_Physcomitrella patens
OS07G02140_Oryza sativa ssp. japonica
SM00022G00950_Selaginella moellendorffii
AT1G12130_Arabidopsis thaliana
AT1G62560_Arabidopsis thaliana
XP_795759_Strongylocentrotus purpuratus
NP_610217_Drosophila melanogaster
XP_001743805_Monosiga brevicollis MX1
OT18G01270_Ostreococcus tauri
EOD12832_Emiliania huxleyi CCMP1516
SB02G031950_Sorghum bicolor
ZM04G02390_Zea mays
ZM07G21490_Zea mays
SB02G031940_Sorghum bicolor
SB02G004310_Sorghum bicolor
RC30178G00380_Ricinus communis
RC30170G06750_Ricinus communis
TC09G006430_Theobroma cacao
TC09G005930_Theobroma cacao
EOD28543_Emiliania huxleyi CCMP1516
type I FMOs
OQD90612_Penicillium antarcticum
XP_016250389_Cladophialophora immunda
KXS21491_Gonapodya prolifera JEL478
EWZ01236_Fusarium oxysporum NRRL 32931
WP_042337516_Bacillus andreraoultii
WP_010571230_Leptospira broomii
ORY08256_Basidiobolus meristosporus CBS 931.73
ADY57965_Rubinisphaera brasiliensis DSM 5305
WP_098503826_Thermoflexus hugenholtzii
EOD36290_Emiliania huxleyi CCMP1516
BVMOs
OHZ38954_Agrobacterium vitis
KDE39435_Nitrincola lacisaponensis
OSX95564_Bacillus mycoides
CUV18971_Ralstonia solanacearum
Q5W9R9_Pseudomonas sp.
class G FMOs
0.1

(b)

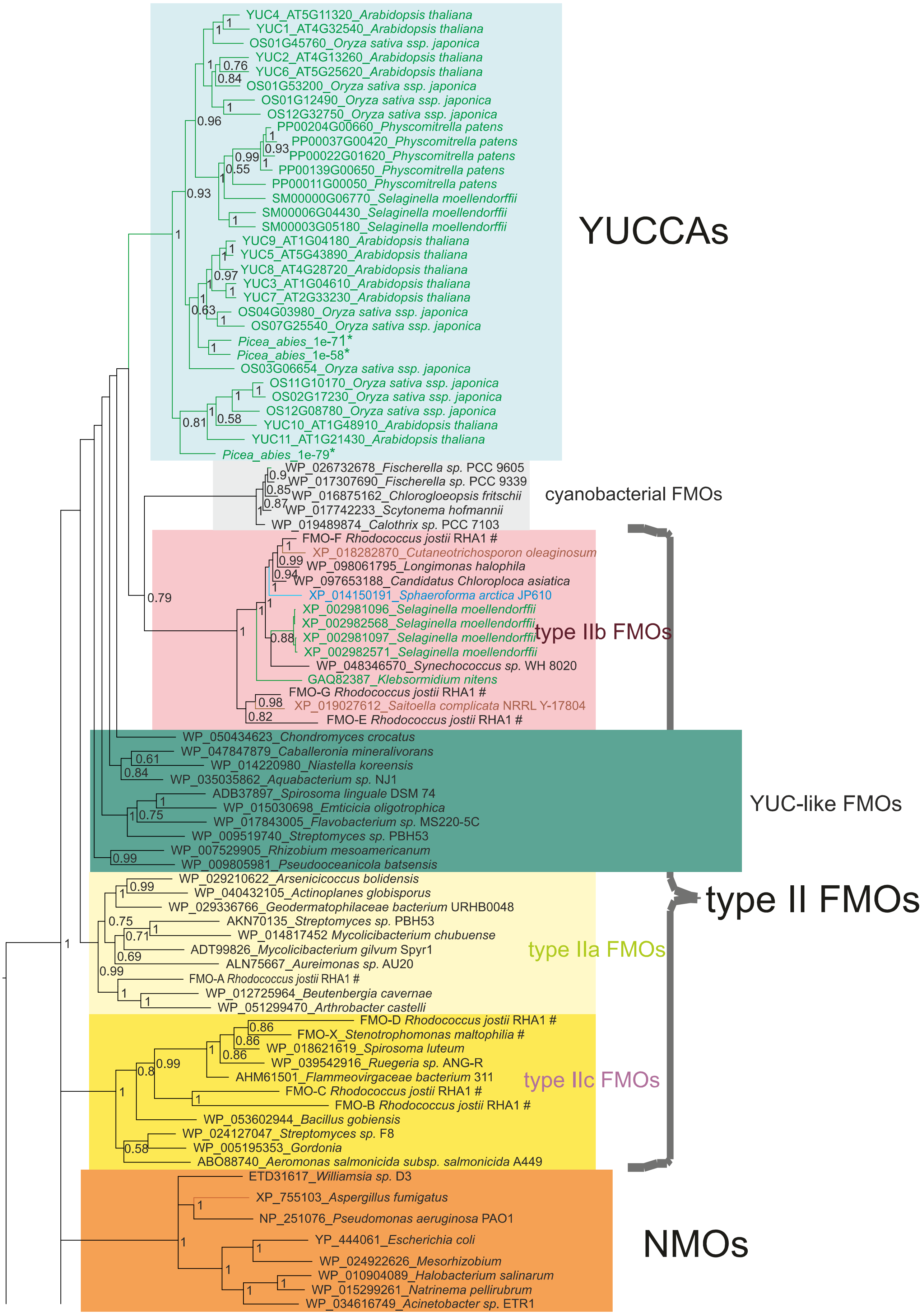
YUCCAs
YUC4_AT5G11320_Arabidopsis thaliana
YUC1_AT4G32540_Arabidopsis thaliana
OS01G45760_Oryza sativa ssp. japonica
YUC2_AT4G13260_Arabidopsis thaliana
YUC6_AT5G25620_Arabidopsis thaliana
OS01G53200_Oryza sativa ssp. japonica
OS01G12490_Oryza sativa ssp. japonica
OS12G32750_Oryza sativa ssp. japonica
PP00204G00660_Physcomitrella patens
PP00037G00420_Physcomitrella patens
PP00022G01620_Physcomitrella patens
PP00139G00650_Physcomitrella patens
PP00011G00050_Physcomitrella patens
SM00000G06770_Selaginella moellendorffii
SM00006G04430_Selaginella moellendorffii
SM00003G05180_Selaginella moellendorffii
YUC9_AT1G04180_Arabidopsis thaliana
YUC5_AT5G43890_Arabidopsis thaliana
YUC8_AT4G28720_Arabidopsis thaliana
YUC3_AT1G04610_Arabidopsis thaliana
YUC7_AT2G33230_Arabidopsis thaliana
OS04G03980_Oryza sativa ssp. japonica
OS07G25540_Oryza sativa ssp. japonica
Picea_abies_1e-71*
Picea_abies_1e-58*
OS03G06654_Oryza sativa ssp. japonica
OS11G10170_Oryza sativa ssp. japonica
OS02G17230_Oryza sativa ssp. japonica
OS12G08780_Oryza sativa ssp. japonica
YUC10_AT1G48910_Arabidopsis thaliana
YUC11_AT1G21430_Arabidopsis thaliana
Picea_abies_1e-79*
cyanobacterial FMOs
WP_026732678_Fischerella sp. PCC 9605
WP_017307690_Fischerella sp. PCC 9339
WP_016875162_Chlorogloeopsis fritschii
WP_017742233_Scytonema hofmannii
WP_019489874_Calothrix sp. PCC 7103
type IIb FMOs
FMO-F Rhodococcus jostii RHA1 #
XP_018282870_Cutaneotrichosporon oleaginosum
WP_098061795_Longimonas halophila
WP_097653188_Candidatus Chloroploca asiatica
XP_014150191_Sphaeroforma arctica JP610
XP_002981096_Selaginella moellendorffii
XP_002982568_Selaginella moellendorffii
XP_002981097_Selaginella moellendorffii
XP_002982571_Selaginella moellendorffii
WP_048346570_Synechococcus sp. WH 8020
GAQ82387_Klebsormidium nitens
FMO-G Rhodococcus jostii RHA1 #
XP_019027612_Saitoella complicata NRRL Y-17804
FMO-E Rhodococcus jostii RHA1 #
YUC-like FMOs
WP_050434623_Chondromyces crocatus
WP_047847879_Caballeronia mineralivorans
WP_014220980_Niastella koreensis
WP_035035862_Aquabacterium sp. NJ1
ADB37897_Spirosoma linguale DSM 74
WP_015030698_Emticicia oligotrophica
WP_017843005_Flavobacterium sp. MS220-5C
WP_009519740_Streptomyces sp. PBH53
WP_007529905_Rhizobium mesoamericanum
WP_009805981_Pseudooceanicola batsensis
type II FMOs
type IIa FMOs
WP_029210622_Arsenicicoccus bolidensis
WP_040432105_Actinoplanes globisporus
WP_029336766_Geodermatophilaceae bacterium URHB0048
AKN70135_Streptomyces sp. PBH53
WP_014817452 Mycolicibacterium chubuense
ADT99826_Mycolicibacterium gilvum Spyr1
ALN75667_Aureimonas sp. AU20
FMO-A Rhodococcus jostii RHA1 #
WP_012725964_Beutenbergia cavernae
WP_051299470_Arthrobacter castelli
type IIc FMOs
FMO-D Rhodococcus jostii RHA1 #
FMO-X_Stenotrophomonas maltophilia #
WP_018621619_Spirosoma luteum
WP_039542916_Ruegeria sp. ANG-R
AHM61501_Flammeovirgaceae bacterium 311
FMO-C Rhodococcus jostii RHA1 #
FMO-B Rhodococcus jostii RHA1 #
WP_053602944_Bacillus gobiensis
WP_024127047_Streptomyces sp. F8
WP_005195353_Gordonia
ABO88740_Aeromonas salmonicida subsp. salmonicida A449
NMOs
ETD31617_Williamsia sp. D3
XP_755103_Aspergillus fumigatus
NP_251076_Pseudomonas aeruginosa PAO1
YP_444061_Escherichia coli
WP_024922626_Mesorhizobium
WP_010904089_Halobacterium salinarum
WP_015299261_Natrinema pellirubrum
WP_034616749_Acinetobacter sp. ETR1

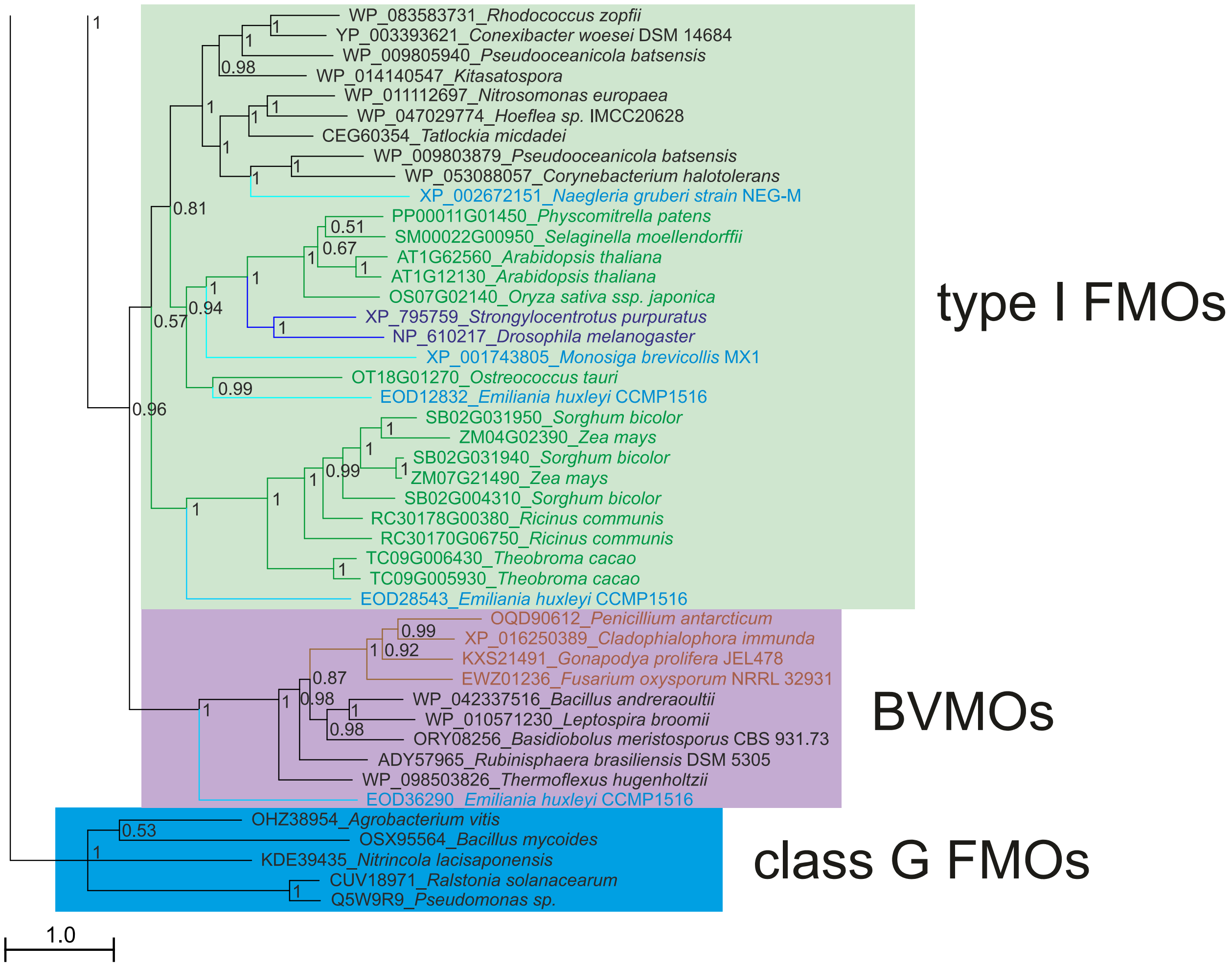

Figure S1. Phylogenetic tree of the class B flavoprotein monooxygenases, class_B_FMO_proteins sample. The branches of green alga and land plant proteins are green; of fungi, brown; of protists, cyan; of animals, blue; of bacteria, black; and of archaebacteria, gray. The class G flavoprotein monooxygenase proteins is outgroup (dark blue background). # denotes protein sequences from Riebel et al. [ref. 40 of the main text]: FMO-A – FMO-G from *R. jostii* RHA1; FMO-X *S. maltophilia* (B2FLR2). Asterisk (*) denotes proteins identified in the Spruce Genome Project [refs. 67,68 of the main text; Congenie.org]. (a) Phylogeny reconstructed using RAxML. The numbers near the branches represent bootstrap support values. (b) Phylogeny reconstructed using mrBayes. The numbers near the branches represent posterior probability values.

(a)

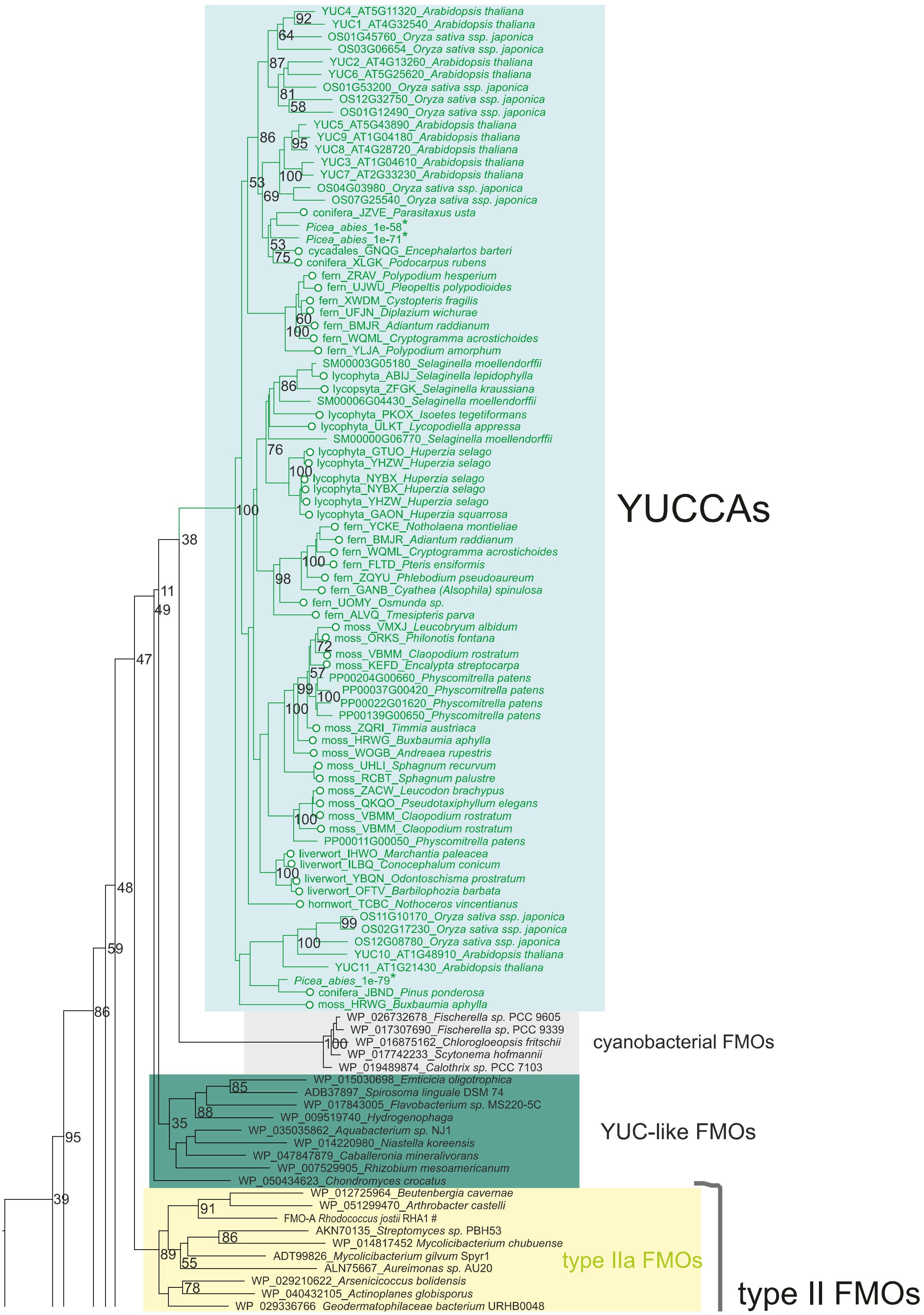
YUC4_AT5G11320_Arabidopsis thaliana
YUC1_AT4G32540_Arabidopsis thaliana
OS01G45760_Oryza sativa ssp. japonica
OS03G06654_Oryza sativa ssp. japonica
YUC2_AT4G13260_Arabidopsis thaliana
YUC6_AT5G25620_Arabidopsis thaliana
OS01G53200_Oryza sativa ssp. japonica
OS12G32750_Oryza sativa ssp. japonica
OS01G12490_Oryza sativa ssp. japonica
YUC5_AT5G43890_Arabidopsis thaliana
YUC9_AT1G04180_Arabidopsis thaliana
YUC8_AT4G28720_Arabidopsis thaliana
YUC3_AT1G04610_Arabidopsis thaliana
YUC7_AT2G33230_Arabidopsis thaliana
OS04G03980_Oryza sativa ssp. japonica
OS07G25540_Oryza sativa ssp. japonica
conifera_JZVE_Parasitaxus usta
Picea_abies_1e-58*
Picea_abies_1e-71*
cycadales_GNQG_Encephalartos barteri
conifera_XLGK_Podocarpus rubens
fern_ZRAV_Polypodium hesperium
fern_UJWU_Pleopeltis polypodioides
fern_XWDM_Cystopteris fragilis
fern_UFJN_Diplazium wichurae
fern_BMJR_Adiantum raddianum
fern_WQML_Cryptogramma acrostichoides
fern_YLJA_Polypodium amorphum
SM00003G05180_Selaginella moellendorffii
lycophyta_ABIJ_Selaginella lepidophylla
lycopsyta_ZFGK_Selaginella kraussiana
SM00006G04430_Selaginella moellendorffii
lycophyta_PKOX_Isoetes tegetiformans
lycophyta_ULKT_Lycopodiella appressa
SM00000G06770_Selaginella moellendorffii
lycophyta_GTUO_Huperzia selago
lycophyta_YHZW_Huperzia selago
lycophyta_NYBX_Huperzia selago
lycophyta_NYBX_Huperzia selago
lycophyta_YHZW_Huperzia selago
lycophyta_GAON_Huperzia squarrosa
fern_YCKE_Notholaena montieliae
fern_BMJR_Adiantum raddianum
fern_WQML_Cryptogramma acrostichoides
fern_FLTD_Pteris ensiformis
fern_ZQYU_Phlebodium pseudoaureum
fern_GANB_Cyathea (Alsophila) spinulosa
fern_UOMY_Osmunda sp.
fern_ALVQ_Tmesipteris parva
moss_VMXJ_Leucobryum albidum
moss_ORKS_Philonotis fontana
moss_VBMM_Claopodium rostratum
moss_KEFD_Encalypta streptocarpa
PP00204G00660_Physcomitrella patens
PP00037G00420_Physcomitrella patens
PP00022G01620_Physcomitrella patens
PP00139G00650_Physcomitrella patens
moss_ZQRI_Timmia austriaca
moss_HRWG_Buxbaumia aphylla
moss_WOGB_Andreaea rupestris
moss_UHLI_Sphagnum recurvum
moss_RCBT_Sphagnum palustre
moss_ZACW_Leucodon brachypus
moss_QKQO_Pseudotaxiphyllum elegans
moss_VBMM_Claopodium rostratum
moss_VBMM_Claopodium rostratum
PP00011G00050_Physcomitrella patens
liverwort_IHWO_Marchantia paleacea
liverwort_ILBQ_Conocephalum conicum
liverwort_YBQN_Odontoschisma prostratum
liverwort_OFTV_Barbilophozia barbata
hornwort_TCBC_Nothoceros vincentianus
OS11G10170_Oryza sativa ssp. japonica
OS02G17230_Oryza sativa ssp. japonica
OS12G08780_Oryza sativa ssp. japonica
YUC10_AT1G48910_Arabidopsis thaliana
YUC11_AT1G21430_Arabidopsis thaliana
Picea_abies_1e-79*
conifera_JBND_Pinus ponderosa
moss_HRWG_Buxbaumia aphylla
YUCCAs
WP_026732678_Fischerella sp. PCC 9605
WP_017307690_Fischerella sp. PCC 9339
WP_016875162_Chlorogloeopsis fritschii
WP_017742233_Scytonema hofmannii
WP_019489874_Calothrix sp. PCC 7103
cyanobacterial FMOs
WP_015030698_Emticicia oligotrophica
ADB37897_Spirosoma linguale DSM 74
WP_017843005_Flavobacterium sp. MS220-5C
WP_009519740_Hydrogenophaga
WP_035035862_Aquabacterium sp. NJ1
WP_014220980_Niastella koreensis
WP_047847879_Caballeronia mineralivorans
WP_007529905_Rhizobium mesoamericanum
WP_050434623_Chondromyces crocatus
YUC-like FMOs
WP_012725964_Beutenbergia cavernae
WP_051299470_Arthrobacter castelli
FMO-A Rhodococcus jostii RHA1 #
AKN70135_Streptomyces sp. PBH53
WP_014817452 Mycolicibacterium chubuense
ADT99826_Mycolicibacterium gilvum Spyr1
ALN75667_Aureimonas sp. AU20
WP_029210622_Arsenicicoccus bolidensis
WP_040432105_Actinoplanes globisporus
WP_029336766_Geodermatophilaceae bacterium URHB0048
type IIa FMOs
type II FMOs

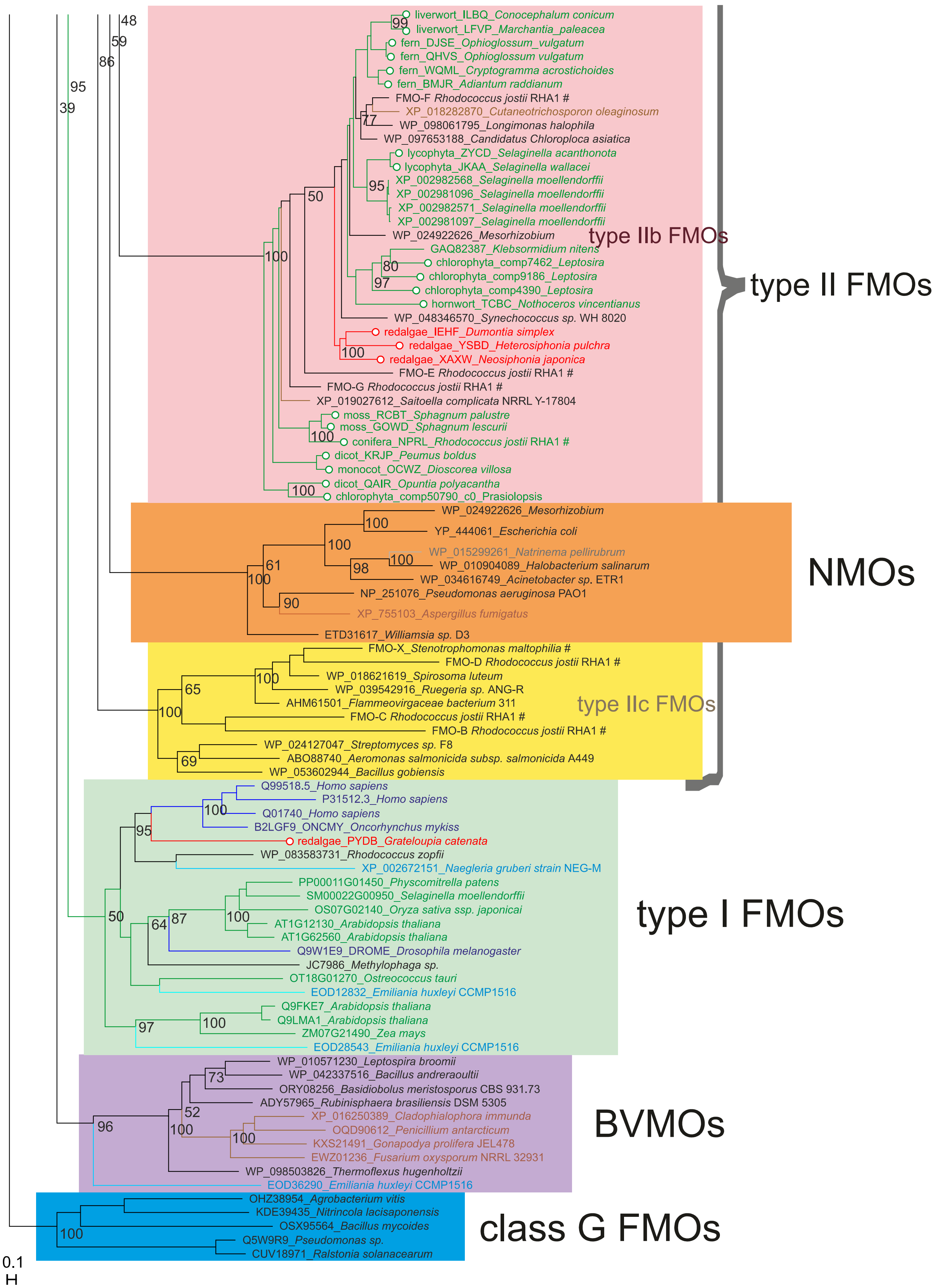

liverwort_ILBQ_Conocephalum conicum
liverwort_LFVP_Marchantia_paleacea
fern_DJSE_Ophioglossum_vulgatum
fern_QHVS_Ophioglossum vulgatum
fern_WQML_Cryptogramma acrostichoides
fern_BMJR_Adiantum raddianum
FMO-F Rhodococcus jostii RHA1 #
XP_018282870_Cutaneotrichosporon oleaginosum
WP_098061795_Longimonas halophila
WP_097653188_Candidatus Chloroploca asiatica
lycophyta_ZYCD_Selaginella acanthonota
lycophyta_JKAA_Selaginella wallacei
XP_002982568_Selaginella moellendorffii
XP_002981096_Selaginella moellendorffii
XP_002982571_Selaginella moellendorffii
XP_002981097_Selaginella moellendorffii
WP_024922626_Mesorhizobium
type IIb FMOs
GAQ82387_Klebsormidium nitens
chlorophyta_comp7462_Leptosira
chlorophyta_comp9186_Leptosira
chlorophyta_comp4390_Leptosira
hornwort_TCBC_Nothoceros vincentianus
WP_048346570_Synechococcus sp. WH 8020
redalgae_IEHF_Dumontia simplex
redalgae_YSBD_Heterosiphonia pulchra
redalgae_XAXW_Neosiphonia japonica
FMO-E Rhodococcus jostii RHA1 #
FMO-G Rhodococcus jostii RHA1 #
XP_019027612_Saitoella complicata NRRL Y-17804
moss_RCBT_Sphagnum palustre
moss_GOWD_Sphagnum lescurii
conifera_NPRL_Rhodococcus jostii RHA1 #
dicot_KRJP_Peumus boldus
monocot_OCWZ_Dioscorea villosa
dicot_QAIR_Opuntia polyacantha
chlorophyta_comp50790_c0_Prasiolopsis
type II FMOs
WP_024922626_Mesorhizobium
YP_444061_Escherichia coli
WP_015299281_Natrinema pellirubrum
WP_010904089_Halobacterium salinarum
WP_034616749_Acinetobacter sp. ETR1
NP_251076_Pseudomonas aeruginosa PAO1
XP_755103_Aspergillus fumigatus
ETD31617_Williamsia sp. D3
NMOs
FMO-X_Stenotrophomonas maltophilia #
FMO-D Rhodococcus jostii RHA1 #
WP_018621619_Spirosoma luteum
WP_039542916_Ruegeria sp. ANG-R
AHM61501_Flammeovirgaceae bacterium 311
type IIc FMOs
FMO-C Rhodococcus jostii RHA1 #
FMO-B Rhodococcus jostii RHA1 #
WP_024127047_Streptomyces sp. F8
ABO88740_Aeromonas salmonicida subsp. salmonicida A449
WP_053602944_Bacillus gobiensis
Q99518.5_Homo sapiens
P31512.3_Homo sapiens
Q01740_Homo sapiens
B2LGF9_ONCMY_Oncorhynchus mykiss
redalgae_PYDB_Grateloupia catenata
WP_083583731_Rhodococcus zopfii
XP_002672151_Naegleria gruberi strain NEG-M
PP00011G01450_Physcomitrella patens
SM00022G00950_Selaginella moellendorffii
OS07G02140_Oryza sativa ssp. japonicai
AT1G12130_Arabidopsis thaliana
AT1G62560_Arabidopsis thaliana
Q9W1E9_DROME_Drosophila melanogaster
JC7986_Methylophaga sp.
OT18G01270_Ostreococcus tauri
EOD12832_Emiliania huxleyi CCMP1516
Q9FKE7_Arabidopsis thaliana
Q9LMA1_Arabidopsis thaliana
ZM07G21490_Zea mays
EOD28543_Emiliania huxleyi CCMP1516
type I FMOs
WP_010571230_Leptospira broomii
WP_042337516_Bacillus andreraoultii
ORY08256_Basidiobolus meristosporus CBS 931.73
ADY57965_Rubinisphaera brasiliensis DSM 5305
XP_016250389_Cladophialophora immunda
OQD90612_Penicillium antarcticum
KXS21491_Gonapodya prolifera JEL478
EWZ01236_Fusarium oxysporum NRRL 32931
WP_098503826_Thermoflexus hugenholtzii
EOD36290_Emiliania huxleyi CCMP1516
BVMOs
OHZ38954_Agrobacterium vitis
KDE39435_Nitrincola lacisaponensis
OSX95564_Bacillus mycoides
Q5W9R9_Pseudomonas sp.
CUV18971_Ralstonia solanacearum
class G FMOs
0.1

(b)

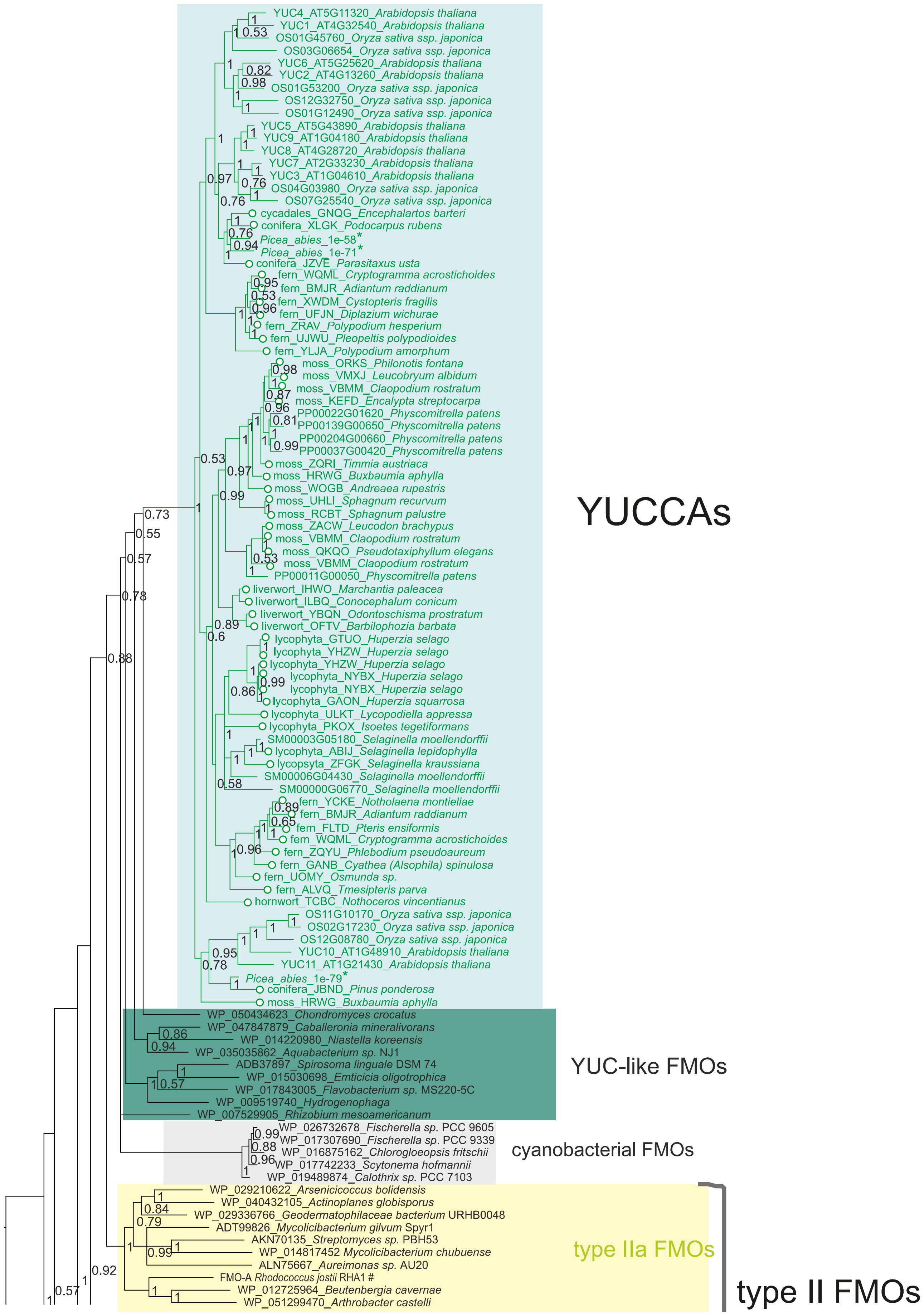

YUC4_AT5G11320_Arabidopsis thaliana
YUC1_AT4G32540_Arabidopsis thaliana
OS01G45760_Oryza sativa ssp. japonica
OS03G06654_Oryza sativa ssp. japonica
YUC6_AT5G25620_Arabidopsis thaliana
YUC2_AT4G13260_Arabidopsis thaliana
OS01G53200_Oryza sativa ssp. japonica
OS12G32750_Oryza sativa ssp. japonica
OS01G12490_Oryza sativa ssp. japonica
YUC5_AT5G43890_Arabidopsis thaliana
YUC9_AT1G04180_Arabidopsis thaliana
YUC8_AT4G28720_Arabidopsis thaliana
YUC7_AT2G33230_Arabidopsis thaliana
YUC3_AT1G04610_Arabidopsis thaliana
OS04G03980_Oryza sativa ssp. japonica
OS07G25540_Oryza sativa ssp. japonica
cycadales_GNQG_Encephalartos barteri
conifera_XLGK_Podocarpus rubens
Picea_abies_1e-58*
Picea_abies_1e-71*
conifera_JZVE_Parasitaxus usta
fern_WQML_Cryptogramma acrostichoides
fern_BMJR_Adiantum raddianum
fern_XWDM_Cystopteris fragilis
fern_UFJN_Diplazium wichurae
fern_ZRAV_Polypodium hesperium
fern_UJWU_Pleopeltis polypodioides
fern_YLJA_Polypodium amorphum
moss_ORKS_Philonotis fontana
moss_VMXJ_Leucobryum albidum
moss_VBMM_Claopodium rostratum
moss_KEFD_Encalypta streptocarpa
PP00022G01620_Physcomitrella patens
PP00139G00650_Physcomitrella patens
PP00204G00660_Physcomitrella patens
PP00037G00420_Physcomitrella patens
moss_ZQRI_Timmia austriaca
moss_HRWG_Buxbaumia aphylla
moss_WOGB_Andreaea rupestris
moss_UHLI_Sphagnum recurvum
moss_RCBT_Sphagnum palustre
moss_ZACW_Leucodon brachypus
moss_VBMM_Claopodium rostratum
moss_QKQO_Pseudotaxiphyllum elegans
moss_VBMM_Claopodium rostratum
PP00011G00050_Physcomitrella patens
liverwort_IHWO_Marchantia paleacea
liverwort_ILBQ_Conocephalum conicum
liverwort_YBQN_Odontoschisma prostratum
liverwort_OFTV_Barbilophozia barbata
lycophyta_GTUO_Huperzia selago
lycophyta_YHZW_Huperzia selago
lycophyta_YHZW_Huperzia selago
lycophyta_NYBX_Huperzia selago
lycophyta_NYBX_Huperzia selago
lycophyta_GAON_Huperzia squarrosa
lycophyta_ULKT_Lycopodiella appressa
lycophyta_PKOX_Isoetes tegetiformans
SM00003G05180_Selaginella moellendorffii
lycophyta_ABIJ_Selaginella lepidophylla
lycopsyta_ZFGK_Selaginella kraussiana
SM00006G04430_Selaginella moellendorffii
SM00000G06770_Selaginella moellendorffii
fern_YCKE_Notholaena montieliae
fern_BMJR_Adiantum raddianum
fern_FLTD_Pteris ensiformis
fern_WQML_Cryptogramma acrostichoides
fern_ZQYU_Phlebodium pseudoaureum
fern_GANB_Cyathea (Alsophila) spinulosa
fern_UOMY_Osmunda sp.
fern_ALVQ_Tmesipteris parva
hornwort_TCBC_Nothoceros vincentianus
OS11G10170_Oryza sativa ssp. japonica
OS02G17230_Oryza sativa ssp. japonica
OS12G08780_Oryza sativa ssp. japonica
YUC10_AT1G48910_Arabidopsis thaliana
YUC11_AT1G21430_Arabidopsis thaliana
Picea_abies_1e-79*
conifera_JBND_Pinus ponderosa
moss_HRWG_Buxbaumia aphylla
YUCCAs
WP_050434623_Chondromyces crocatus
WP_047847879_Caballeronia mineralivorans
WP_014220980_Niastella koreensis
WP_035035862_Aquabacterium sp. NJ1
ADB37897_Spirosoma linguale DSM 74
WP_015030698_Emticicia oligotrophica
WP_017843005_Flavobacterium sp. MS220-5C
WP_009519740_Hydrogenophaga
WP_007529905_Rhizobium mesoamericanum
YUC-like FMOs
WP_026732678_Fischerella sp. PCC 9605
WP_017307690_Fischerella sp. PCC 9339
WP_016875162_Chlorogloeopsis fritschii
WP_017742233_Scytonema hofmannii
WP_019489874_Calothrix sp. PCC 7103
cyanobacterial FMOs
WP_029210622_Arsenicicoccus bolidensis
WP_040432105_Actinoplanes globisporus
WP_029336766_Geodermatophilaceae bacterium URHB0048
ADT99826_Mycolicibacterium gilvum Spyr1
AKN70135_Streptomyces sp. PBH53
WP_014817452 Mycolicibacterium chubuense
ALN75667_Aureimonas sp. AU20
FMO-A Rhodococcus jostii RHA1 #
WP_012725964_Beutenbergia cavernae
WP_051299470_Arthrobacter castelli
type IIa FMOs
type II FMOs

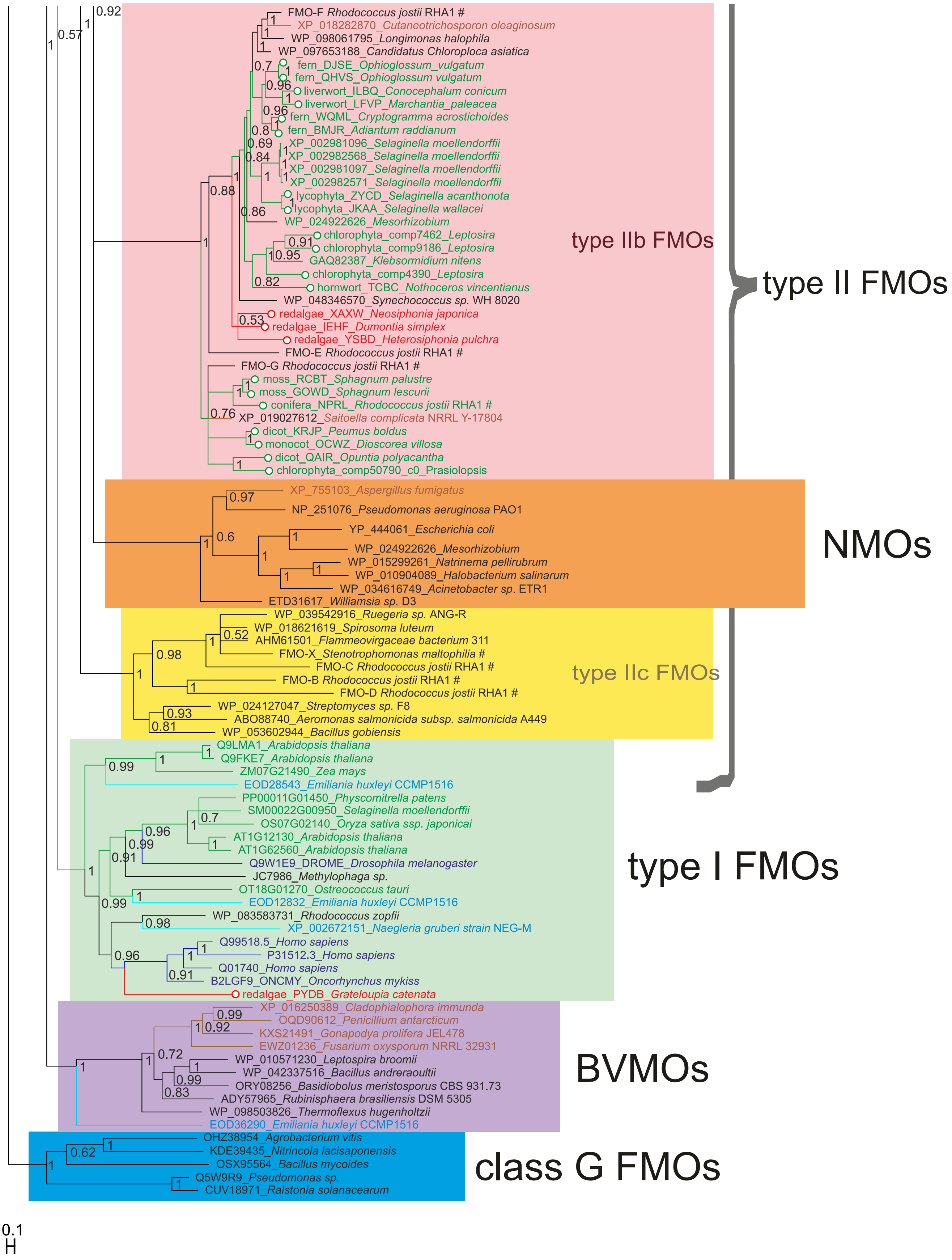

Figure S2. Phylogenetic trees of the class B flavoprotein monooxygenases, class_B_FMO_proteins_ext sample. All designations are as in Figure S1a; sequences from the 1KP project [ref. 49 of the main text] and the Green Algal Tree of Life Project [ref. 26 of the main text] are marked with a circle. The sequences of green algae and land plants are colored green; of red algae, red; of fungi, brown; of protists, cyan; of animals, blue; of bacteria, black, and of archaebacteria, gray. The class G flavoprotein

monooxygenase proteins is outgroup (dark blue background). # denotes protein sequences from Riebel et al. [ref. 17 of the main text]: FMO-A – FMO-G from *R. jostii* RHA1; FMO-X *S. maltophilia* (B2FLR2). Asterisk (*) denotes proteins identified in the Spruce Genome Project [refs. 67,68 of the main text; Congenie.org]. (a) Phylogeny reconstructed using RAxML. The numbers near the branches represent bootstrap support values. (b) Phylogeny reconstructed using mrBayes. The numbers near the branches represent posterior probability values.

(a)

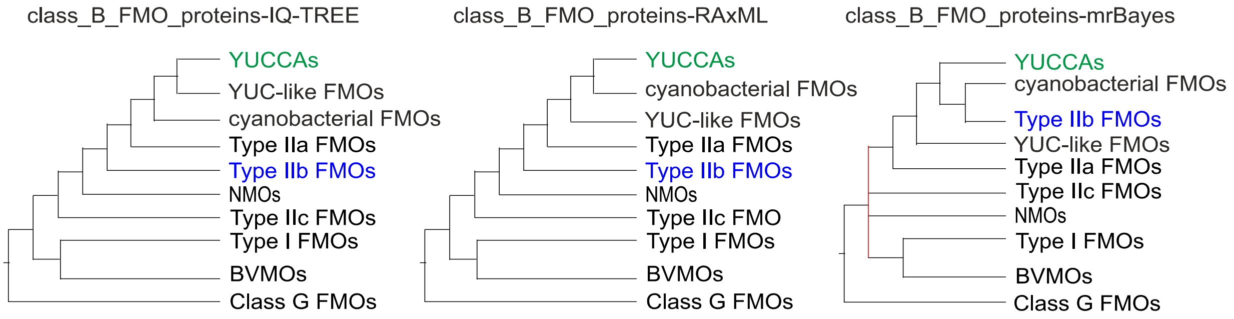

(b)

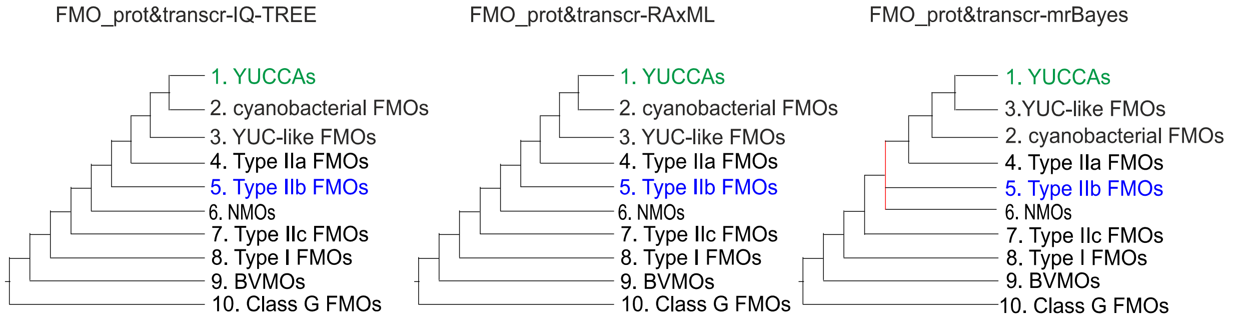

Figure S3. Simplified cladograms of the phylogenetic trees of the class B flavoprotein monooxygenases constructed by: IQ-TREE, RAxML and mrBayes. The YUCCA clade is denoted with green and type IIb FMO clade, with dark blue. Red line denotes a polyfurcations.

(a) Cladograms of the trees built using class B flavoprotein monooxygenase proteins (class_B_FMO_proteins_ext sample).

(b) Cladograms of the trees built using extended class B flavoprotein monooxygenase proteins (class_B_FMO_proteins_ext sample).
